# Thiourea-Derived Chelating Ligands and Their Organometallic Compounds: Investigations into Their Anticancer Activity

**DOI:** 10.3390/molecules25163661

**Published:** 2020-08-11

**Authors:** Kelvin K. H. Tong, Muhammad Hanif, James H. Lovett, Katja Hummitzsch, Hugh H. Harris, Tilo Söhnel, Stephen M. F. Jamieson, Christian G. Hartinger

**Affiliations:** 1 School of Chemical Sciences, University of Auckland, Private Bag 92019, Auckland 1142, New Zealand; kton030@aucklanduni.ac.nz (K.K.H.T.); m.hanif@auckland.ac.nz (M.H.); t.soehnel@auckland.ac.nz (T.S.); 2 Department of Chemistry, The University of Adelaide, Adelaide, SA 5005, Australia; james.lovett@student.adelaide.edu.au (J.H.L.); hugh.harris@adelaide.edu.au (H.H.H.); 3 Discipline of Obstetrics and Gynecology, The University of Adelaide, Robinson Research Institute, Adelaide, SA 5005, Australia; katja.hummitzsch@adelaide.edu.au; 4 Auckland Cancer Society Research Centre, University of Auckland, Private Bag 92019, Auckland 1142, New Zealand; s.jamieson@auckland.ac.nz

**Keywords:** bioorganometallics, cancer chemotherapeutics, hydrogen bonds, molecular structures, thione ligands, X-ray fluorescence microscopy

## Abstract

Thiones have been investigated as ligands in metal complexes with catalytic and biological activity. We report the synthesis, characterization, and biological evaluation of a series of M^II/III^ complexes of the general formulae [M^II^(cym)(L)Cl]X (cym = η^6^-*p*-cymene) or [M^III^(Cp*)(L)Cl]X (Cp* = η^5^-pentamethylcyclopentadienyl), where X = Cl^−^ or PF_6_^−^, and L represents heterocyclic derivatives of thiourea. The thiones feature a benzyl-triazolyl pendant and they act as bidentate ligands via *N*,*S*-coordination to the metal centers. Several derivatives have been investigated by single-crystal X-ray diffraction analysis. NMR investigations showed a counterion-dependent shift of several protons due to the interaction with the counterions. These NMR investigations were complemented with X-ray diffraction analysis data and the effects of different counterions on the secondary coordination sphere were also investigated by DFT calculations. In biological studies, the Ir benzimidazole derivative was found to accumulate in the cytoplasm and it was the most cytotoxic derivative investigated.

## 1. Introduction

Ligand design has been shown to be a key aspect in the development of metal complexes for biological applications ranging from MRI contrast agents to anticancer drugs [1]. Classic coordination compounds and more recently organometallic analogues have been investigated for their tumor-inhibiting properties with the ligands often strongly influencing their biological activity and characteristics such as pharmacokinetics [1,2]. This has led to the development of a wide variety of metal-based anticancer agents following the success of the commonly used Pt drugs with all their shortcomings [3,4,5,6,7].

Half-sandwich organometallics are some of the most widely investigated compounds. Initially being led by Ru(arene) derivatives with their designable biological properties [6,8,9,10,11,12,13,14], more and more isostructural Os analogues as well as Rh- and Ir(Cp*) derivatives with interesting properties have been reported [13,15,16]. The differences in properties can often be pinned down to varying ligand exchange kinetics, target site specificity and lipophilicity [13,17,18,19,20,21,22], which allow for more efficient interactions with biological target molecules, binding at specific target sites, and cell accumulation, respectively. However, in all these complexes, ligand choice and design are key to obtain compounds with desirable biological properties and low toxicity.

Imidazole is found as a building block of molecules in nature and it features in a wide range of biologically active compounds [23,24], with applications ranging from antiparasitic to anticancer activity. Its use as an important scaffold in medicinal chemistry has led to a detailed understanding of its chemistry and the preparation of many derivatives. In medicinal inorganic chemistry, imidazole moieties provide a versatile platform for ligand design both in terms of the coordination ability and access to structural modifications. Functional groups can be introduced, for example, through alkylation of one or both endocyclic nitrogen atoms, the latter leading to *N*-heterocyclic carbene (NHC) ligand precursors, which are widely used for applications, such as, catalysis, and have more recently also been investigated for biological properties when coordinated to metal centers [25]. The imidazolium-based NHC precursors can also be converted into thiourea derivatives [26], featuring a soft S-donor for metal coordination. Such thiones are good σ-donors and weak π-acceptors and a wide range of metal complexes have been synthesized, often for investigations into their catalytic properties [26,27,28,29,30,31]. As with thiones [32,33,34,35], some of their metal complexes have shown biological activity. For example, we and others reported on the anticancer activity of organometallic derivatives [36,37,38,39,40,41,42], some of which were derived from biologically active ligands such as the antibacterial agent nalidixic acid [37].

Herein, we report the preparation of a series of thione complexes from ligands prepared in click reactions, as well as investigations into their stability, distribution in cells, and potency in in vitro anticancer activity assays.

## 2. Results and Discussion

**Scheme 1:**
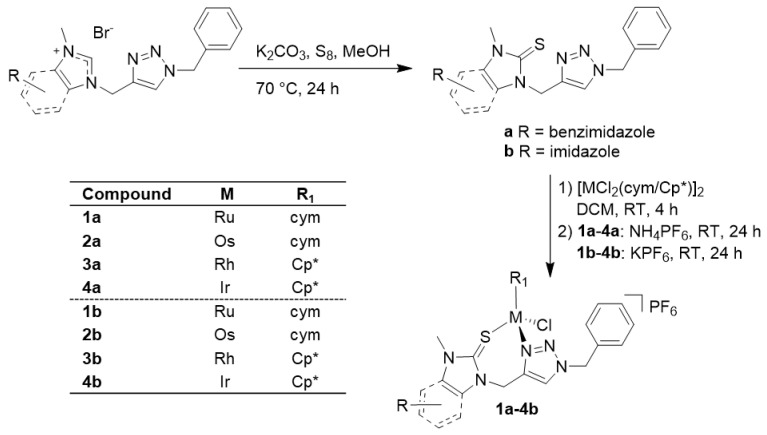
Synthesis of the thione ligands and their metal complexes **1a**–**4b**.

The thione ligands **a** and **b** were prepared by the thionation of 1-[(1-benzyl-1,2,3-triazol-4-yl)methyl]-3-methylimidazolium bromide or 1-[(1-benzyl-1,2,3-triazol-4-yl)methyl]-3-methylbenzimidazolium bromide, respectively, using potassium carbonate as a base (Scheme 1) following a procedure reported for similar compounds [26]. Both **a** and **b** were obtained in moderately high yields as white solids and the identities of **a** and **b** were confirmed by ^1^H and ^13^C{^1^H} NMR spectroscopy, electrospray ionization mass spectrometry (ESI-MS), and elemental analysis. Conversion of the imidazolium group into a thione resulted in a characteristic peak at about 10 ppm being absent in the NMR spectra of **a** and **b**. In the ^13^C{^1^H}-NMR spectra of **a** and **b**, singlets at about 170 and 160 ppm, respectively, were observed which confirmed the formation of the C=S group. The loss of the cationic nature of the imidazolium moiety significantly changed the chemical shifts of the signals of the surrounding protons such as of the methyl, methylene, and triazolyl groups. ESI-MS gave mainly pseudomolecular [M + Na]^+^ ions for ligands **a** and **b**.

By reacting the thione ligands with the metal dimers [M^II/III^(cym/Cp*)Cl_2_]_2_ (where M = Ir^III^, Os^II^, Rh^III^, or Ru^II^; cym = *p*-cymene; Cp* = pentamethylcyclopentadiene), along with the appropriate hexafluorophosphate salts for counterion metathesis (NH_4_PF_6_ or KPF_6_; *vide infra*), the corresponding cationic metal complexes **1a**–**4b** were obtained in moderate to high yields (61–96%; Scheme 1). The presence of the thione and the triazole groups allowed **a** and **b** to act as bidentate *N*,*S*-donor systems. The metal complexes were characterized by NMR spectroscopy, elemental analysis, and/or ESI-MS. In some cases, quaternary carbon atoms were not detected in the ^13^C{^1^H}-NMR spectra and the values were used from 2D NMR experiments. Upon metal coordination, the proton signals of the ligand shifted significantly. The ^1^H-NMR spectra of the **1a**–**4b** indicated a downfield shift of the triazolyl proton signal from 7.8 to 8.5 ppm, depending on the metal center. The deshielding effect experienced by the triazolyl and methyl proton signals slightly increased as the metal center became heavier. Coordination of the ligands to the metal centers resulted in the signals of the methylene protons in vicinity of the 7-membered metalla-ring to become diastereotopic. Geminal coupling of these protons was observed in the ^1^H-NMR spectra at approximately 15 Hz, with the protons resonating in the range of 5.2 to 5.9 ppm. For the imidazole-thione complexes **1b**–**4b**, the signals of the methylene group bridging the triazolyl and benzyl groups also exhibited geminal coupling with similar coupling constants. The aromatic cym protons of the Ru complexes **1a** and **1b** overlapped with the methylene proton signals. In the ^13^C{^1^H}-NMR spectra of all complexes, the signal attributed to the C=S group and the quaternary carbon of the triazolyl group experienced an upfield shift upon metal coordination. The Cp* carbon atoms coordinated to Rh resonated as a doublet due to C–Rh coupling (^1^*J* = 8 Hz). The identity of the complexes was further confirmed by ESI-MS studies. The ESI-mass spectra of complexes **1a**–**4b** featured pseudomolecular [M – PF_6_]^+^ ions at *m*/*z* values closely resembling the calculated values. Furthermore, peak assignment was supported by the characteristic isotope pattern for the complexes. Elemental analysis data indicated the presence of solvent molecules in small traces for some compounds and these solvents were evident in the ^1^H-NMR spectra and considered when calculating the yields.

Single crystals suitable for X-ray diffraction analysis of **3b** and **4a** were grown via slow diffusion of toluene into a saturated solution of the complexes in acetonitrile and they crystallized in the orthorhombic and monoclinic space groups *Pna*2_1_ and *P*2_1_/*c*, respectively (Supporting Information). The pseudo-octahedral configuration and the characteristic “piano-stool” scaffold were observed for all complexes, where the Cp* (**3b**, **4a**) acted as the seat along with the *N*,*S*-chelating thiourea-triazolyl and chlorido ligands as the legs of the stool (Figure S1, Supporting Information). The coordination of the bidentate ligands to the metal centers resulted in the establishment of 7-membered metalla-ring systems with slightly distorted boat conformation. The complexes crystallized as enantiomeric mixtures with the metal centers being stereogenic. In the Ir complex **4a**, the Ir–N_triazole_ bond was slightly shorter than found in **3b** (Table 1), while there was hardly any difference between the imidazole- and benzimidazole-derived complexes, also in terms of M–S bond length.

**Figure 1:**
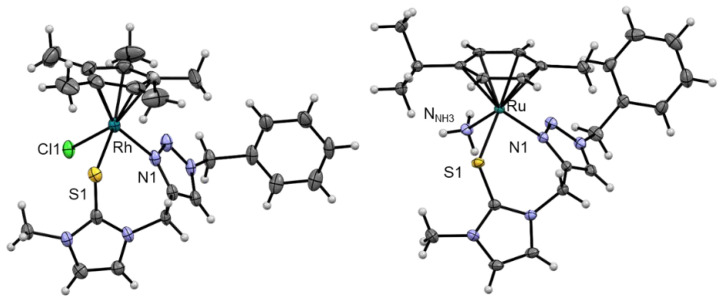
Molecular structures of one of the enantiomers found for **3b** (**left**), and [Ru^II^(cym)(NH_3_)(**b**)](PF_6_)_2_ **1b^NH3^** (**right**). Counterions and co-crystallized solvent molecules have been omitted for clarity and the structures are drawn at 50% probability level.

The counterion metathesis reactions were successful for the syntheses of **1a**–**4a** with NH_4_PF_6_ as the PF_6_^−^ source. However, **1b** could not be prepared with NH_4_PF_6_ as indicated by two sets of signals in the ^1^H-NMR spectrum at a ratio of 0.3:1. The impurity was identified by X-ray diffraction analysis of a single crystal obtained from the NMR sample (CDCl_3_) as the ammine complex [Ru^II^(cym)(NH_3_)(**b**)](PF_6_)_2_ (**1b^NH3^**; Figure 1). The data revealed that the chlorido ligand of **1b** was substituted with ammine, resulting in a doubly charged complex cation and two PF_6_^−^ counter ions. It appears that the ammine ligand originated from the excess of NH_4_PF_6_ added to the reaction mixture. The formation of [Ru^II^(cym)(NH_3_)(**b**)](PF_6_)_2_ was further confirmed by ESI-MS in which the pseudomolecular ion [{Ru^II^(cym)(NH_3_)(**b**)}PF_6_]^+^ was detected at *m*/*z* 683.1122 (*m*/*z*_theor_ 683.1095). To prevent the undesirable NH_3_/Cl^−^ ligand exchange during the reaction, KPF_6_ was employed instead to successfully synthesize **1b**–**4b** in high purity.

In addition to the complexes with PF_6_^−^ as the counterion, the chloride derivatives **1a^Cl^**–**4b^Cl^** were synthesized in similar yields (56–96%) using the same procedures without adding the hexafluorophosphate salts in the last step for metathesis (Scheme 1). Single crystals suitable for X-ray diffraction analyses of **2a^Cl^**, **3b^Cl^**, and **4b^Cl^** were grown via slow diffusion of toluene into a saturated solution of the complexes in acetonitrile. Complexes **3b^Cl^** and **4b^Cl^** crystallized in the triclinic space group *P*-1 and **2a^Cl^** in monoclinic *P*2_1_/*n* (Supporting Information). Both enantiomers were found in the molecular structures which were overall very similar to those of the analogous complexes with hexafluorophosphate as the counterion (Table 1). The M–Cp*_centroid_ distance in **2a^Cl^**, however, was significantly shorter than found for the Cp* complexes [43].

**Figure 2:**
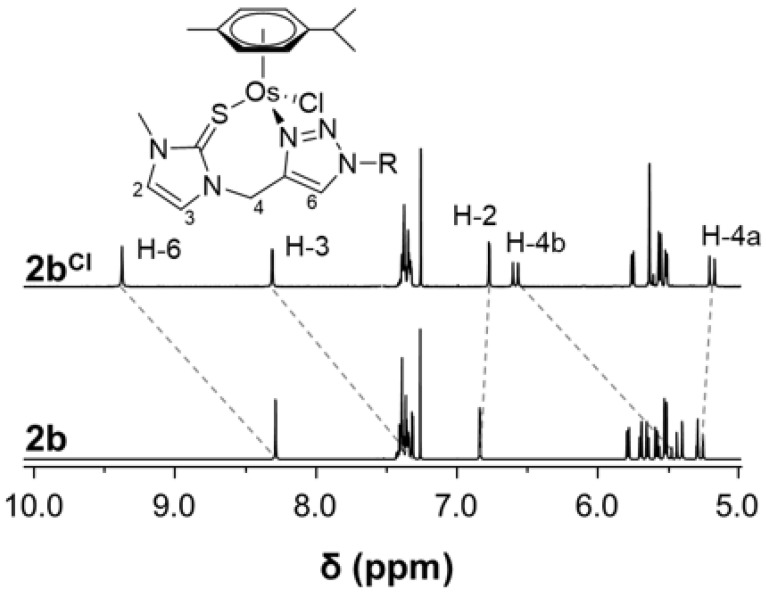
Comparison of the aromatic regions of the ^1^H-NMR spectra of **2b** with its PF_6_^−^ counterion and of the chloride derivative **2b^Cl^** recorded in CDCl_3_.

Interestingly, the ^1^H-NMR spectra of the PF_6_^−^ and Cl^−^ derivatives showed that the chemical shifts of several proton signals were affected by the change in counterion (Figure 2). These signals corresponded to the triazolyl, the methylene, and the benzimidazole or imidazole protons as part of the 7-membered metalla-ring system. The respective protons of the Cl^−^ derivatives were typically detected about 1.0 ppm downfield compared to their PF_6_^−^ counterparts, while the chemical shifts of other proton signals, i.e., of the cym and benzyl groups, remained considerably unaffected. We attribute this effect to the differing ability of the counterions to interact with the hydrogen atoms surrounding the metal centers.

**Figure 3:**
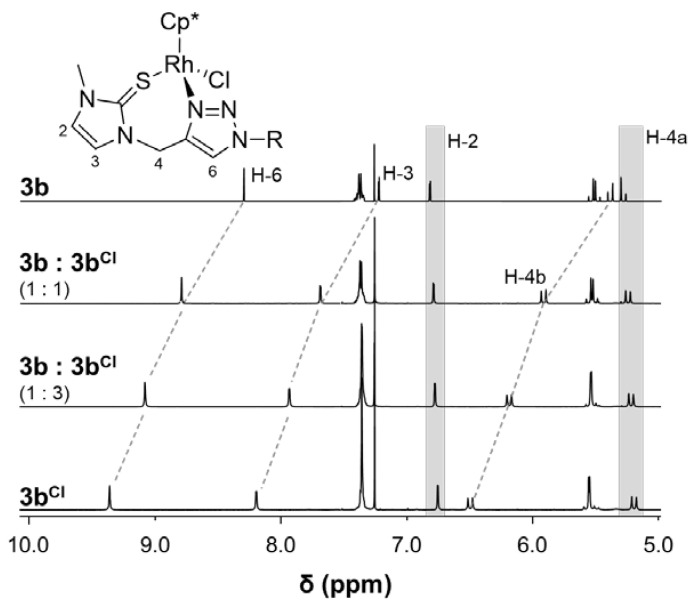
The aromatic regions of the ^1^H-NMR spectra of **3b** with its PF_6_^−^ counterion and the chloride derivative **3b^Cl^**, and both compounds mixed at 1:1 and 1:3 ratios.

To investigate this effect further, **3b** and **3b^Cl^** were analyzed in CDCl_3_ by ^1^H-NMR spectroscopy in comparison to solutions containing both of **3b** and **3b^Cl^** at 1:1 and 1:3 ratios (Figure 3). In all ^1^H-NMR spectra, only one set of signals was observed, which indicates that the complex cations of **3b** and **3b^Cl^** are identical. The signals of three protons, i.e., H-3, H-4, and H-6, experienced significant changes in their chemical shifts, when comparing the isolated compounds and the mixtures, while the other protons resonated virtually at the same frequencies. By increasing the amount of **3b^Cl^**, the downfield shifts were more pronounced with the signals obtained for **3b** at highest field and for **3b^Cl^** at lowest. This suggests an interaction of the protons H-3, H-4, and H-6, which form a slight pocket according to their molecular structures, with the counterions present in solution. A similar experiment was conducted with **4a** and **4a^Cl^**, which were selected for their benzimidazole group and therewith a structural change of the pocket formed next to the triazole moiety. As with **3b** and **3b^Cl^**, the signals of some of the triazole, methylene, and benzimidazole protons shifted significantly downfield (Supporting Information) upon increasing the concentration of **4a^Cl^** in the solution. However, when the same samples of **3b** and **3b^Cl^** were prepared in MeOD, the differences in chemical shifts of these protons were no longer observed (Supporting Information). The results suggest that the presence of PF_6_^−^ and Cl^−^ ions in CDCl_3_ solution could potentially establish hydrogen bonding interactions with the protons of the complexes next to the triazole moiety which are absent in protic solvents such as methanol.

**Figure 4:**
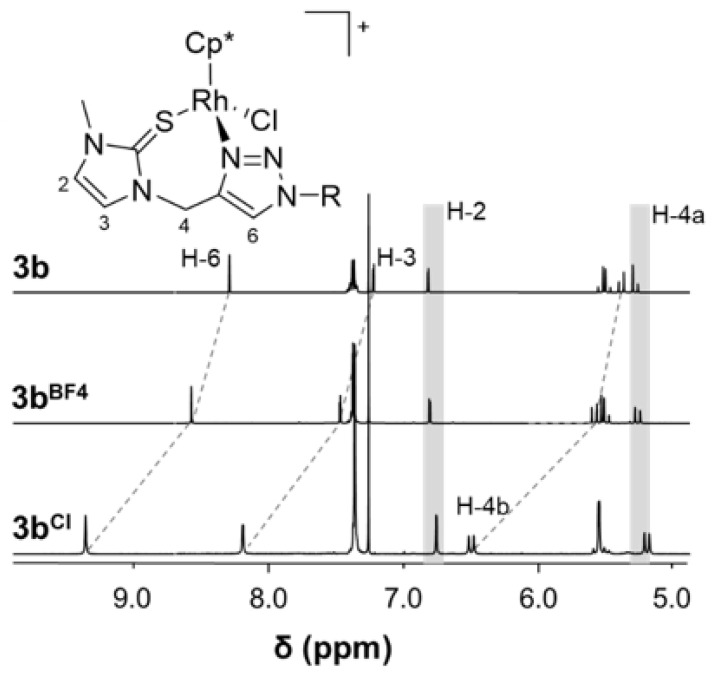
Comparison of the aromatic regions of the ^1^H-NMR spectra of **3b** with its PF_6_^−^ counterion and of the tetrafluoroborate **3b^BF4^** and chloride **3b^Cl^** derivatives.

Similar observations were made when the counterion was replaced with BF_4_^−^. Complex **3b^BF4^** was obtained in a counterion metathesis reaction from **3b^Cl^** suspended in a methanol solution containing 10 molar equiv. of NaBF_4_. The ^1^H-NMR spectrum of **3b^BF4^** showed that the H-6 signal was shifted downfield by 0.3 ppm relative to the same signal of **3b** (Figure 4, Table 2). The trend observed for the chloride, tetrafluoroborate and hexafluorophosphate counterions followed their hydrogen bond formation ability as determined for 1-butyl-3-methylimidazolium salts [44], and consequently the imidazolium proton is detected at highest field for the PF_6_^−^ salt followed by BF_4_^−^ and Cl^−^.

Density functional theory (DFT) calculations were then implemented to investigate the secondary coordination sphere by simulating the ^1^H-NMR spectra of **3b**, **3b^Cl^**, and **3b^BF4^** based on the molecular structures. The optimized structures revealed the distances between the three protons in the thione/triazole pocket which shifted significantly in the NMR spectroscopy investigations, i.e., H-3, H-4b, and H-6, and the counterions were within the plausible range of establishing hydrogen bond interactions. In general, the chemical shifts of the proton signals obtained by simulation of the ^1^H-NMR spectra were considerably similar to those observed in the recorded ^1^H-NMR spectra (Table 2). Both the simulated and experimental results support that the chemical shifts of H-3, H-4, and H-6 can be influenced by the counterion present in a non-protic solvent system.

**Figure 5:**
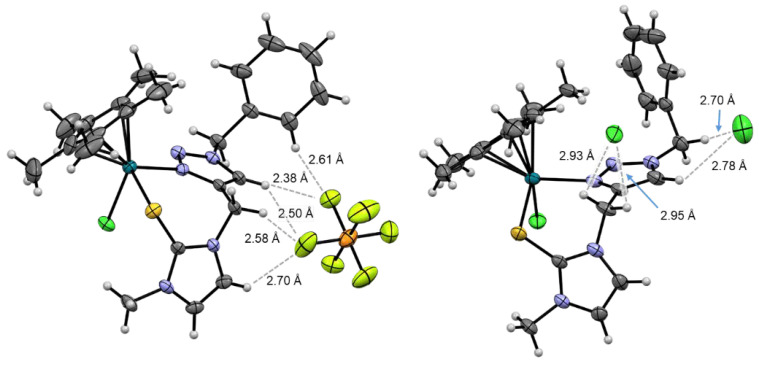
Interactions between the PF_6_^−^ counterions and the complex cations in **3b** (**left**) and **3b^Cl^** (**right**) with the C–H···X (X = Cl or PF_6_) distances shown. Co-crystallized solvent molecules have been omitted for clarity and the structures are drawn at 50% probability level.

Revisiting the molecular structures of the complexes with PF_6_^−^ counterions revealed structural differences between the imidazole and benzimidazole derivatives **3b** and **4a** (Figure 5 and Supporting Information). In case of **3b**, the counteranion interacts through C–H···F_6_P hydrogen bonds with the triazole, CH_2,NHC_, phenyl, and imidazole protons. In **4a**, the steric constraint caused by the introduction of the benzimidazole moiety leads to interactions with the triazole, CH_2,Bn_, phenyl, and benzimidazole protons and the CH_2,NHC_ protons, with a second PF_6_^−^ ion sitting on top of the bidentate ligand (Supporting Information). A CH_2,Bn_ proton in **3b** however forms another hydrogen bond with a second PF_6_^−^ positioned on the opposite side of the ligand. When comparing the structures of the PF_6_^−^ complexes with those with Cl^−^ counterions, the chloride anions interact with the complex cations very differently and the C–H···X (X = Cl or PF_6_) bond lengths are longer for the complexes with Cl^−^ (Figure 5). They are part of a hydrogen bonding network involving co-crystallized water molecules (data not shown). In **2a^Cl^**, one of the chloride ions interacts with the triazole proton while in **3b^Cl^** in addition H bonding with the CH_2,Bn_ protons was observed (Figure 5 and Supporting Information). The differences in C–H···X interactions support the data obtained in the NMR studies where the protons in the pocket formed from thione and triazole moieties shifted significantly in dependence of the ability of the counterions to form hydrogen bonds.

Organometallic compounds, especially those which feature metal–halido bonds, often undergo ligand exchange reactions when present in solutions of water, or DMSO. To study the in vitro anticancer activity, compounds are usually dissolved in DMSO before they are diluted with cell culture growth medium. Therefore, the stability of the metal complexes in different solvents was investigated by ^1^H-NMR spectroscopy. The Ru complexes **1a** and **1a^Cl^** and Ir compounds **4a** and **4a^Cl^** were investigated for their stability in DMSO-*d*_6_ solutions. Both compounds gave very different time-dependent ^1^H-NMR spectra which we attribute to the observation described above for the dependence of the chemical shift of some of the protons on the counterions. Therefore, the spectra of **4a^Cl^** gave a single set of signals, however, after rapid exchange of the chlorido ligand with DMSO. For **4a** with its PF_6_^−^ counterion, substitution of the chlorido ligands for DMSO resulted in two main sets of signals, one of which resonated at the same frequencies as in **4a^Cl^** while others may be due to further ligand exchange reactions (Supporting Information). The imidazole-thione derivatives **1b**–**4b** were investigated on their aqueous stability in 15% DMSO-*d*_6_/D_2_O by ^1^H-NMR spectroscopy over a period of 120 h. For all complexes studied, the signals remained unchanged throughout the experiment indicating sufficient stability for biological investigations (Supporting Information).

The in vitro anticancer activity of the hexafluorophosphate complexes **1a**–**4b** was studied against human colorectal (HCT116), non-small-cell lung (NCI-H460), cervical carcinoma (SiHa), and colon adenocarcinoma (SW480) cancer cells. In general, the imidazole-thione derivatives were considered inactive with IC_50_ values > 100 μM, while some of the thione complexes demonstrated moderate activity (Table 3). The Ir complex **4a** was the most potent antiproliferative agent within the series of complexes with IC_50_ values as low as 20 and 25 μM against HCT116 and NCI-H460 cells, respectively, while it was less active against SiHa and SW480 cells. Complexes **1a**–**3a** showed some activity in HCT116 cells and organo-osmium compound **2a** also in NCI-H460 but these did not reach the potency of the Ir analogue. These observations suggest that slower ligand exchange kinetics in this complex type is key to high cytotoxic activity. Furthermore, the lower lipophilicity of the imidazole- as compared to the benzimidazole-derived compounds explains the inactivity of **1b**–**4b**.

**Figure 6:**
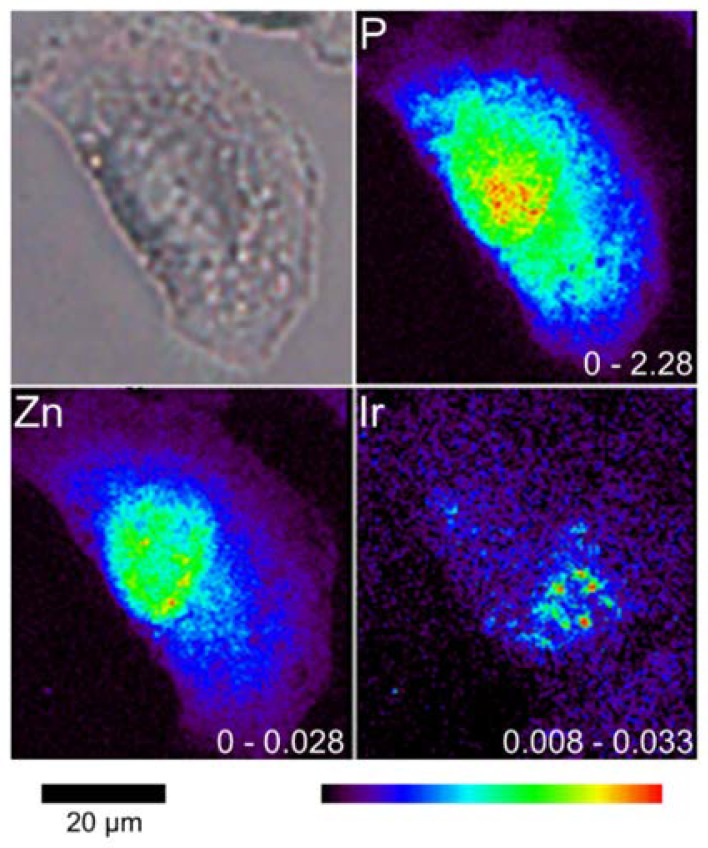
P, Zn, and Ir distribution (heatmap color scale) in SKOV-3 cancer cells after treatment with **4a** as determined by X-ray fluorescence microscopy. The number ranges in the bottom right corners indicate fluorescence counts as areal densities (μg cm^−2^).

Since **4a** was the most potent anticancer agent in the in vitro cytotoxicity studies, it was selected for further investigations into the uptake and localization in SKOV-3 cells by X-ray fluorescence microscopy (XFM). The cell nuclei can be distinguished in XFM studies due to the high quantities of Zn and P present. While the controls did not feature any signal for Ir (data not shown), the cells treated with **4a** showed significant amounts of the compound had been taken up into the cells (Figure 6). When the distribution of Zn and Ir in the cells were compared, no co-localization was observed. This indicated that **4a** accumulates in SKOV-3 cells in the cell cytoplasm rather than in the nuclei where they could interact with nuclear DNA. Therefore, it is unlikely that DNA is the molecular target of the thione complexes.

## 3. Materials and Methods

All air-sensitive reactions were carried out under an N_2_ flow in standard Schlenk or round-bottom flasks. The preparation of the complexes was done in darkness by covering the reaction apparatus with aluminum foil to prevent photolytic degradation. Dichloromethane was dried prior to use with Na_2_SO_4_, while all other solvents purchased from commercial suppliers were used without further purification. 1-Methylbenzimidazole (AK-Scientific, Union City, CA, USA, 98%), 1-methylimidazole (Acros Organics, Fair Lawn, NJ, USA, 99%), 1,2,3,4,5-pentamethylcyclopentadiene (Merck, Darmstadt, Germany, ≥88%) ammonium hexafluorophosphate (Acros Organics, Fair Lawn, NJ, USA, 99%), α-terpinene (Sigma-Aldrich, St. Louis, MO, USA, 89%), benzyl bromide (Merck, Darmstadt, Germany, ≥98%), copper sulfate pentahydrate (ECP, Auckland, New Zealand, ≥≥98.0%), iridium(III) chloride hydrate (Precious Metal Online, Wollongong, Australia, 99%), osmium(III) chloride hydrate (Heraeus South Africa, Port Elizabeth, South Africa, 55% metal content), potassium carbonate (ECP, Auckland, New Zealand, ≥99.9%), potassium hexafluorophosphate (Sigma-Aldrich, St. Louis, MO, USA, ≥99.0%), propargyl bromide (Aldrich, St. Louis, MO, USA, 80 wt.% in toluene), rhodium(III) chloride hydrate (Precious Metal Online, Wollongong, Australia, 99%), ruthenium(III) chloride hydrate (Precious Metal Online, Wollongong, Australia, 99%), sodium azide (Sigma-Aldrich, St. Louis, MO, USA, ≥99%), sodium l-ascorbate (Sigma-Aldrich, St. Louis, MO, USA, ≥98%), sodium sulfate anhydrous (ECP, Auckland, New Zealand, ≥98%), and sulfur (Sigma-Aldrich, St. Louis, MO, USA) were from commercial suppliers and used as received. The precursors [OsCl_2_(η^6^-*p*-cymene)]_2_ [45,46], [RuCl_2_(η^6^-*p*-cymene)]_2_ [47], [IrCl_2_(η^5^-pentamethylcyclopentadiene)]_2_ [48] and [RhCl_2_(η^5^-pentamethylcyclopentadiene)]_2_ [49] were synthesized following literature procedures. 1-[(1-Benzyl-1,2,3-triazol-4-yl)methyl]-3-methylimidazolium bromide and 1-[(1-benzyl-1,2,3-triazol-4-yl)methyl]-3-methylbenzimidazolium bromide were prepared according to ref. [50] using THF: H_2_O = 1:1 as the solvent instead of DMSO.

### 3.1. Physical Measurements

Elemental analyses were conducted on a vario EL cube (Elementar Analysensysteme GmbH, Hanau, Germany). Both 1D (^1^H, ^13^C{^1^H}DEPT-Q, and ^31^P{^1^H}) and 2D (^1^H−^1^H COSY, ^1^H−^13^C HSQC, and ^1^H−^13^C HMBC) NMR spectra were recorded on Bruker Avance AVIII 400 MHz NMR spectrometers at 400.13 MHz (^1^H) or 100.57 MHz (^13^C{^1^H}) at ambient temperature. CDCl_3_, DMSO-*d_6_*, and D_2_O were used as NMR solvents. Electrospray ionization mass spectra were recorded on a Bruker micrO-TOF-Q II ESI-MS in positive ion mode.

X-ray diffraction measurements of single crystals of **1b^NH3^, 2a^Cl^**, **3b**, **3b^Cl^**, **4a**, and **4b^Cl^** were performed on a Rigaku Oxford Diffraction XtaLAB-Synergy-S single-crystal diffractometer (Rigaku Corporation, Tokyo, Japan) with a PILATUS 200K hybrid pixel array detector using Cu Kα radiation (λ = 1.54184 Å; Supporting Information). The data were processed with the SHELX2016/6 [51] and Olex2 [52,53] software packages. All non-hydrogen atoms were refined anisotropically. Hydrogen atoms were inserted at calculated positions and refined with a riding model or without restrictions. Mercury 4.3.1 was used to visualize the molecular structures [54].

### 3.2. Syntheses

General Procedure of the Synthesis of Ligands **a** and **b**.

1-[(1-Benzyl-1,2,3-triazol-4-yl)methyl]-3-methylimidazolium bromide or 1-[(1-benzyl-1,2,3-triazol-4-yl)methyl]-3-methylbenzimidazolium bromide (1.0 mol equiv.) and K_2_CO_3_ (2.0 mol equiv.) were dissolved in methanol and sulfur (1.1 mol equiv.) was added. The reaction mixture was refluxed for 24 h. The solvent was removed, and the product was extracted with dichloromethane (3 × 100 mL) and water (100 mL). The organic layer was collected and dried over anhydrous Na_2_SO_4_, followed by evaporation of the solvent to obtain the pure products as white solids.

*1-[(1-Benzyl-1,2,3-triazol-4-yl)methyl]-3-methylbenzimidazole-2-thione***a**, The synthesis of **a** was performed according to the general procedure using 1-[(1-benzyl-1,2,3-triazol-4-yl)methyl]-3-methylbenzimidazolium bromide (0.50 g, 1.3 mmol), K_2_CO_3_ (0.36 g, 2.6 mmol), and sulfur (0.046 g, 1.4 mmol) to afford a white solid (0.22 g, 73%). Calcd. for C_18_H_17_N_5_S·0.15H_2_O: C, 63.94; H, 5.16; N, 20.71%. Found: C, 63.84; H, 4.94; N, 20.63%. MS (ESI^+^): *m*/*z* = 358.1106 [M + Na]^+^ (m_calc_ = 358.1102). ^1^H-NMR (399.89 MHz, CDCl_3_); δ (ppm) 7.74 (s, 1H, H-10), 7.60–7.56 (m, 1H, H-3/H-6), 7.37–7.31 (m, 3H, H-13, H-14, H-15), 7.25–7.20 (m, 4H, H-4, H-5, H-12, H-16), 7.16–7.12 (m, 1H, H-3/H-6), 5.63 (s, 2H, H-8), 5.43 (s, 2H, H-11), 3.77 (s, 3H, H-1). ^13^C{^1^H}-NMR (100.57 MHz, CDCl_3_); δ (ppm) 169.33 (C-18), 142.9 (C-9), 134.3 (C-17), 132.4 (C-2), 131.5 (C-7), 129.1 (C-12/C-13/C-14/C-15/C-16), 128.8 (C-12/C-13/C-14/C-15/C-16), 128.1 (C-12/C-13/C-14/C-15/C-16), 123.3 (C-4/C-5/C-10), 123.2 (C-4/C-5/C-10), 110.3 (C-3/C-6), 108.8 (C-3/C-6), 54.2 (C-11), 40.0 (C-8), 31.3 (C-1).

*1-[(1-Benzyl-1,2,3-triazol-4-yl)methyl]-3-methylimidazole-2-thione***b**, The synthesis of **b** was performed according to the general procedure using 1-[(1-benzyl-1,2,3-triazol-4-yl)methyl]-3-methylimidazolium bromide (0.45 g, 1.4 mmol), K_2_CO_3_ (0.38 g, 2.7 mmol), and sulfur (0.048 g, 1.5 mmol) to afford a white solid (0.29 g, 75%). Calcd. for C_14_H_15_N_5_S·0.05H_2_O: C, 58.74; H, 5.32; N, 24.46%. Found: C, 58.82; H, 5.39; N, 24.55%. MS (ESI^+^): *m*/*z* = 308.0942 [M + Na]^+^ (m_calc_ = 308.0946). ^1^H-NMR (399.89 MHz, CDCl_3_); δ (ppm) 7.76 (s, 1H, H-6), 7.39–7.33 (m, 3H, H-9, H-10, H-11), 7.27–7.23 (m, 2H, H-8, H-12), 6.91 (d, ^3^*J* = 2.4 Hz, 1H, H-3), 6.63 (d, ^3^*J* = 2.4 Hz, 1H, H-2), 5.47 (s, 2H, H-7), 5.31 (s, 2H, H-4), 3.57 (s, 3H, H-1). ^13^C{^1^H}-NMR (100.57 MHz, CDCl_3_); δ (ppm) 162.2 (C-14), 142.8 C-5), 134.4 (C-13), 129.1 (C-8/C-9/C-10/C-11/C-12), 128.8 (C-8/C-9/C-10/C-11/C-12), 128.1 (C-8/C-9/C-10/C-11/C-12), 123.4 (C-6), 117.9 (C-2), 116.9 (C-3), 54.3 (C-7), 42.7 (C-4), 35.1 (C-1).

General Procedure A for the Preparation of **1a**–**4a** and **1b**–**4b**.

Ligand **a** (2.0 mol equiv.) or **b** (2.0 mol equiv.) was dissolved in dichloromethane, followed by the addition of [M^II/III^Cl_2_(η^6^-*p*-cymene/η^5^-pentamethylcyclopentadiene)]_2_ (1.0 mol equiv.). The reaction mixture was stirred at room temperature for 4 h, ammonium hexafluorophosphate (3 mol equiv.) or potassium hexafluorophosphate (3 mol equiv.) was added and the mixture was stirred for 20 h. The solvent was evaporated, the residue was dissolved in dichloromethane and filtered. The filtrate was concentrated to about 5 mL and diethyl ether (50 mL) was added to induce precipitation. The precipitate was washed with diethyl ether (50 mL) and dried in vacuo to obtain the pure metal complexes.

General Procedure B for the Preparation of **1a^Cl^**–**4a^Cl^** and **7b^C^**–**10b^Cl^**.

Ligand **a** (2.0 mol equiv.) or **b** (2.0 mol equiv.) was dissolved in dichloromethane, followed by the addition of [M^II/III^Cl_2_(η^6^-*p*-cymene/η^5^-pentamethylcyclopentadiene)]_2_ (1.0 mol equiv.). The reaction mixture was stirred at room temperature for 24 h. The solvent was evaporated, the residue was dissolved in dichloromethane and filtered. The filtrate was concentrated to about 5 mL and diethyl ether (50 mL) was added to induce precipitation. The precipitate was washed with additional diethyl ether (50 mL) and dried in vacuo to obtain the pure metal complexes.

*[Chlorido{1-[(1-benzyl-1,2,3-triazol-4-yl-κN)methyl]-3-methylbenzimidazole-2-thione-κS}(η^6^-p-cymene)ruthenium(II)] hexafluorophosphate***1a**, the synthesis of **1a** was performed according to general procedure A using **a** (50 mg, 0.150 mmol), [RuCl_2_(η^6^-*p*-cymene)]_2_ (46 mg, 0.075 mmol), and ammonium hexafluorophosphate (37 mg, 0.220 mmol) to afford an orange powder (70 mg, 61%). Calcd. for C_28_H_31_ClF_6_N_5_PRuS·0.15H_2_O: C, 44.61; H, 4.19; N, 9.29%. Found: C, 44.44; H, 3.96; N, 9.29%. MS (ESI^+^): *m*/*z* = 606.1034 [M – PF_6_]^+^ (m_calc_ = 606.1032). ^1^H-NMR (399.89 MHz, CDCl_3_); δ (ppm) 8.43 (s, 1H, H-10), 7.68 (d, ^3^*J* = 8 Hz, 1H, H-3/H-6), 7.45–7.34 (m, 7H, H-4, H-5, H-12, H-13, H-14, H-15, H-16), 7.30 (d, ^3^*J* = 8 Hz, 1H, H-3/H-6), 5.63–5.57 (m, 2H, H-8a/b, H-21/H-22), 5.52 (s, 2H, H-11), 5.50–5.43 (m, 3H, H-8a/b, H-21/H-22, H-23/H-24), 5.40 (d, ^3^*J* = 6 Hz, 1H, H-23/H-24), 3.87 (s, 3H, H-1), 2.78 (sept, ^3^*J* = 7 Hz, 1H, H-26), 2.08 (s, 3H, H-19), 1.27 (d, ^4^*J* = 7 Hz, 3H, H-27/H-28), 1.21 (d, ^4^*J* = 7 Hz, 3H, H-27/H-28). ^13^C{^1^H}-NMR (100.57 MHz, CDCl_3_); δ (ppm) 163.5 (C-18), 139.2 (C-9), 132.8 (C-17), 132.1 (C-2), 130.7 (C-7), 129.3 (C-12/C-13/C-14/C-15/C-16), 128.9 (C-12/C-13/C-14/C-15/C-16), 126.6 (C-4/C-5), 126.1 (C-4/C-5), 125.6 (C-10), 111.1 (C-3/C-6), 110.8 (C-3/C-6), 105.6 (C-25), 99.2 (C-20), 84.9 (C-21/C-22/C-23/C-24), 84.7 (C-21/C-22/C-23/C-24), 84.6 (C-21/C-22/C-23/C-24), 83.9 (C-21/C-22/C-23/C-24), 55.8 (C-11), 38.4 (C-8), 33.0 (C-1), 30.7 (C-26), 22.1 (C-27/C-28), 18.3 (C-19).

*[Chlorido{1-[(1-benzyl-1,2,3-triazol-4-yl-κN)methyl]-3-methylbenzimidazole-2-thione-κS}(η^6^-p-cymene)ruthenium(II)] hexafluorophosphate***1a**, the synthesis of **1a** was performed according to general procedure A using **a** (50 mg, 0.150 mmol), [RuCl_2_(η^6^-*p*-cymene)]_2_ (46 mg, 0.075 mmol), and ammonium hexafluorophosphate (37 mg, 0.220 mmol) to afford an orange powder (70 mg, 61%). Calcd. for C_28_H_31_ClF_6_N_5_PRuS·0.15H_2_O: C, 44.61; H, 4.19; N, 9.29%. Found: C, 44.44; H, 3.96; N, 9.29%. MS (ESI^+^): *m*/*z* = 606.1034 [M – PF_6_]^+^ (m_calc_ = 606.1032). ^1^H-NMR (399.89 MHz, CDCl_3_); δ (ppm) 8.43 (s, 1H, H-10), 7.68 (d, ^3^*J* = 8 Hz, 1H, H-3/H-6), 7.45–7.34 (m, 7H, H-4, H-5, H-12, H-13, H-14, H-15, H-16), 7.30 (d, ^3^*J* = 8 Hz, 1H, H-3/H-6), 5.63–5.57 (m, 2H, H-8a/b, H-21/H-22), 5.52 (s, 2H, H-11), 5.50–5.43 (m, 3H, H-8a/b, H-21/H-22, H-23/H-24), 5.40 (d, ^3^*J* = 6 Hz, 1H, H-23/H-24), 3.87 (s, 3H, H-1), 2.78 (sept, ^3^*J* = 7 Hz, 1H, H-26), 2.08 (s, 3H, H-19), 1.27 (d, ^4^*J* = 7 Hz, 3H, H-27/H-28), 1.21 (d, ^4^*J* = 7 Hz, 3H, H-27/H-28). ^13^C{^1^H}-NMR (100.57 MHz, CDCl_3_); δ (ppm) 163.5 (C-18), 139.2 (C-9), 132.8 (C-17), 132.1 (C-2), 130.7 (C-7), 129.3 (C-12/C-13/C-14/C-15/C-16), 128.9 (C-12/C-13/C-14/C-15/C-16), 126.6 (C-4/C-5), 126.1 (C-4/C-5), 125.6 (C-10), 111.1 (C-3/C-6), 110.8 (C-3/C-6), 105.6 (C-25), 99.2 (C-20), 84.9 (C-21/C-22/C-23/C-24), 84.7 (C-21/C-22/C-23/C-24), 84.6 (C-21/C-22/C-23/C-24), 83.9 (C-21/C-22/C-23/C-24), 55.8 (C-11), 38.4 (C-8), 33.0 (C-1), 30.7 (C-26), 22.1 (C-27/C-28), 18.3 (C-19).

*[Chlorido{1-[(1-benzyl-1,2,3-triazol-4-yl-κN)methyl]-3-methylbenzimidazole-2-thione-κS}(η^6^-p-cymene)ruthenium(II)] chloride***1a^Cl^**, the synthesis of **1a^Cl^** was performed according to general procedure B using **a** (50 mg, 0.150 mmol) and [RuCl_2_(η^6^-*p*-cymene)]_2_ (46 mg, 0.075 mmol) to afford a brownish red powder (89 mg, 93%). Calcd. for C_28_H_31_Cl_2_N_5_RuS·0.15C_6_H_14_·0.85H_2_O: C, 51.82; H, 5.24; N, 10.45%. Found: C, 51.97; H, 5.08; N, 10.29%. MS (ESI^+^): *m*/*z* = 606.1024 [M – Cl]^+^ (m_calc_ = 606.1032). ^1^H-NMR (399.89 MHz, CDCl_3_); δ (ppm) 9.68 (brs, 1H, H-10), 8.55 (brs, 1H, H-6), 7.47–7.41 (m, 3H, H-4/H-5/H-12/H-13/H-14/H-15/H-16), 7.39–7.30 (m, 4H, H-4/H-5/H-12/H-13/H-14/H-15/H-16), 7.24 (d, ^3^*J* = 8 Hz, 1H, H-3), 6.57 (d, ^2^*J*_H′–H″_ = 15 Hz, 1H, H-8a/b), 5.64 (d, ^2^*J*_H′–H″_ = 14 Hz, 1H, H-11a/b), 5.55–5.37 (m, 5H, H-8a/b, H-11a/b, H-21/H-22/H-23/H-24), 5.32–5.27 (m, 1H, H-21/H-22/H-23/H-24), 3.84 (s, 3H, H-1), 2.67 (sept, ^3^*J* = 7 Hz, 1H, H-26), 1.97 (s, 3H, H-19), 1.24 (d, ^4^*J* = 7 Hz, 3H, H-27/H-28), 1.17 (d, ^4^*J* = 7 Hz, 3H, H-27/H-28). ^13^C{^1^H}-NMR (100.57 MHz, CDCl_3_); δ (ppm) 162.8 (C-18), 139.4 (C-9), 133.7 (C-17), 131.8 (C-2), 131.2 (C-7), 129.0 (C-12/C-13/C-14/C-15/C-16), 128.9 (C-17), 126.3 (C-5), 125.4 (C-4), 113.9 (C-6), 110.2 (C-3), 105.0 (C-25), 98.6 (C-20), 84.9 (C-21/C-22/C-23/C-24), 84.6 (C-21/C-22/C-23/C-24), 84.5 (C-21/C-22/C-23/C-24), 84.0 (C-21/C-22/C-23/C-24), 55.6 (C-11), 39.7 (C-8), 33.0 (C-1), 30.6 (C-26), 22.2 (C-27/C-28), 22.0 (C-27/C-28), 18.3 (C-19).

*[Chlorido{1-[(1-benzyl-1,2,3-triazol-4-yl-κN)methyl]-3-methylbenzimidazole-2-thione-κS}(η^6^-p-cymene)osmium(II)] hexafluorophosphate***2a**, the synthesis of **2a** was performed according to general procedure A using **a** (50 mg, 0.150 mmol), [OsCl_2_(η^6^-*p*-cymene)]_2_ (59 mg, 0.075 mmol), and ammonium hexafluorophosphate (37 mg, 0.220 mmol) to afford a yellow powder (75 mg, 66%). Calcd. for C_28_H_31_ClF_6_N_5_OsPS·1.35H_2_O: C, 38.9; H, 3.93; N, 8.10%. Found: C, 38.66; H, 3.68; N, 8.12%. MS (ESI^+^): *m*/*z* = 696.1576 [M – PF_6_]^+^ (m_calc_ = 696.1603). ^1^H-NMR (399.89 MHz, CDCl_3_); δ (ppm) 8.46 (s, 1H, H-10), 7.75 (d, ^3^*J* = 8 Hz, 1H, H-3/H-6), 7.47 (td, ^3^*J* = 7.5 Hz, ^4^*J* = 1.2 Hz, 1H, H-4/H-5), 7.43–7.32 (m, 7H, H-4/H-5, H-3/H-6, H-12, H-13, H-14, H-15, H-16), 5.85 (d, ^3^*J* = 6 Hz, 1H, H-21/H-22), 5.78 (d, ^3^*J* = 6 Hz, 1H, H-21/H-22), 5.72–5.63 (m, 3H, H-8a/b, H-23, H-24), 5.52–5.47 (m, 3H, H-8a/b, H-11), 3.90 (s, 3H, H-1), 2.67 (sept, ^3^*J* = 7 Hz, 1H, H-26), 2.11 (s, 3H, H-19), 1.26 (d, ^4^*J* = 7 Hz, 3H, H-27/H-28), 1.21 (d, ^4^*J* = 7 Hz, 3H, H-27/H-28). ^13^C{^1^H}-NMR (100.57 MHz, CDCl_3_); δ (ppm) 164.8 (C-18), 137.6 (C-9), 132.7 (C-17), 131.9 (C-2), 130.8 (C-7), 129.4 (C-12/C-13/C-14/C-15/C-16), 129.3 (C-12/C-13/C-14/C-15/C-16), 128.9 (C-12/C-13/C-14/C-15/C-16), 126.5 (C-4/C-5), 126.4 (C-4/C-5), 125.7 (C-10), 111.3 (C-3/C-6), 111.0 (C-3/C-6), 96.6 (C-25), 91.2 (C-20), 77.2 (C-21/C-22/C-23/C-24), 76.5 (C-21/C-22/C-23/C-24), 76.2 (C-21/C-22/C-23/C-24), 76.1 (C-21/C-22/C-23/C-24), 55.8 (C-11), 38.5 (C-8), 33.2 (C-1), 30.7 (C-26), 22.4 (C-27/C28), 22.2 (C-27/C-28), 18.1 (C-19).

*[Chlorido{1-[(1-benzyl-1,2,3-triazol-4-yl-κN)methyl]-3-methylbenzimidazole-2-thione-κS}(η^6^-p-cymene)osmium(II)] chloride***2a^Cl^**, the synthesis of **2a^Cl^** was performed according to the general procedure B using **a** (50 mg, 0.150 mmol) and [OsCl_2_(η^6^-*p*-cymene)]_2_ (59 mg, 0.075 mmol) to afford a yellow powder (61 mg, 56%). MS (ESI^+^): *m*/*z* = 696.1576 [M – Cl]^+^ (m_calc_ = 696.1603). ^1^H-NMR (399.89 MHz, CDCl_3_); δ (ppm) 9.92 (s, 1H, H-10), 8.73 (d, ^3^*J* = 8 Hz, 1H, H-6), 7.50 (td, ^3^*J* = 8 Hz, ^4^*J* = 1 Hz, 1H, H-4/H-5), 7.41–7.33 (m, 6H, H-4/H-5, H-12, H-13, H-14, H-15, H-16), 7.29 (d, ^3^*J* = 8 Hz, 1H, H-3), 6.78 (d, ^2^*J*_H′–H″_ = 15 Hz, 1H, H-8a/b), 5.79 (d, ^3^*J* = 6 Hz, 1H, H-21/H-22), 5.67 (dd, ^3^*J* = 10 Hz, ^4^*J* = 6 Hz, 2H, H-21/H-22, H-23/H-24), 5.59 (d, ^2^*J*_H′–H″_ = 14 Hz, 1H, H-11a/b), 5.54 (d, ^3^*J* = 6 Hz, 1H, H-23/H-24), 5.50 (d, ^2^*J*_H′–H″_ = 14 Hz, 1H, H-11a/b), 5.44 (d, ^2^*J*_H′–H″_ = 15 Hz, 1H, H-8a/b), 3.88 (s, 3H, H-1), 2.58 (sept, ^3^*J* = 7 Hz, 1H, H-26), 2.02 (s, 3H, H-19), 1.22 (d, ^4^*J* = 7 Hz, 3H, H-27/H-28), 1.18 (d, ^4^*J* = 7 Hz, 3H, H-27/H-28). ^13^C{^1^H}-NMR (100.57 MHz, CDCl_3_); δ (ppm) 164.4 (C-18), 137.9 (C-9), 133.4 (C-17), 131.7 (C-2), 131.3 (C-7), 129.1 (C-12/C-13/C-14/C-15/C-16), 128.8 (C-12/C-13/C-14/C-15/C-16), 128.6 (C-10), 126.6 (C-5), 125.5 (C-4), 113.9 (C-6), 110.4 (C-3), 95.8 (C-25), 90.8 (C-20), 76.8 (C-21/C-22/C-23/C-24), 76.4 (C-21/C-22/C-23/C-24), 76.3 (C-21/C-22/C-23/C-24), 75.8 (C-21/C-22/C-23/C-24), 55.5 (C-11), 39.1 (C-8), 33.3 (C-1), 30.7 (C-26), 22.6 (C-27/C-28), 22.1 (C-27/C-28), 18.1 (C-19).

*[Chlorido{1-[(1-benzyl-1,2,3-triazol-4-yl-κN)methyl]-3-methylbenzimidazole-2-thione-κS}(η^5^-pentamethylcyclopentadiene)rhodium(III)] hexafluorophosphate***3a**, the synthesis of **3a** was performed according to general procedure A using **a** (50 mg, 0.150 mmol), [RhCl_2_(η^5^-pentamethylcyclopentadiene)]_2_ (46 mg, 0.075 mmol), and ammonium hexafluorophosphate (37 mg, 0.220 mmol) to afford a red powder (110 mg, 96%). Calcd. for C_28_H_32_ClF_6_N_5_PRhS·0.2H_2_O: C, 44.39; H, 4.31; N, 9.24%. Found: C, 44.53; H, 4.19; N, 9.07%. MS (ESI^+^): *m*/*z* = 608.1125 [M – PF_6_]^+^ (m_calc_ = 608.1122). ^1^H-NMR (399.89 MHz, CDCl_3_); δ (ppm) 8.46 (s, 1H, H-10), 7.69 (d, ^3^*J* = 8 Hz, 1H, H-3/H-6), 7.46 (td, ^3^*J* = 8 Hz, ^4^*J* = 1 Hz, 1H, H-4/H-5), 7.42–7.34 (m, 7H, H-4/H-5, H-12, H-13, H-14, H-15, H-16), 7.32 (d, ^3^*J* = 8 Hz, 1H, H-3/H-6), 5.69 (d, ^2^*J*_H′–H″_ = 16 Hz, 1H, H-8a/b), 5.55–5.47 (m, 3H, H-8a/b, H-11), 3.93 (s, 3H, H-1), 1.57 (s, 15H, H-19). ^13^C{^1^H}-NMR (100.57 MHz, CDCl_3_); δ (ppm) 139.1 (C-9), 133.4 (C-17), 132.8 (C-12), 132.2 (C-7), 129.4 (C-12/C-13/C-14/C-15/C-16), 129.3 (C-12/C-13/C-14/C-15/C-16), 129.1 (C-12/C-13/C-14/C-15/C-16), 126.7 (C-10), 126.2 (C-4/C-5), 125.6 (C-4/C-5), 111.0 (C-3/C-6), 110.8 (C-3/C-6), 96.9 (d, ^1^J(Rh-C_Cp_) = 8 Hz, C-20), 55.9 (C-11), 38.4 (C-8), 32.9 (C-1), 8.9 (C-19).

*[Chlorido{1-[(1-benzyl-1,2,3-triazol-4-yl-κN)methyl]-3-methylbenzimidazole-2-thione-κS}(η^5^-pentamethylcyclopentadiene)rhodium(III)] chloride***3a^Cl^**, the synthesis of **3a^Cl^** was performed according to general procedure B using a (50 mg, 0.150 mmol) and [RhCl_2_(η^5^-pentamethylcyclopentadiene)]_2_ (46 mg, 0.075 mmol) to afford an orange-red powder (88 mg, 92%). MS (ESI^+^): *m*/*z* = 574.1487 [M – PF_6_]^+^ (m_calc_ = 574.1506). ^1^H-NMR (399.89 MHz, CDCl_3_); δ (ppm) 9.85 (s, 1H, H-10), 8.67 (d, ^3^*J* = 8 Hz, 1H, H-6), 7.48 (td, ^3^*J* = 8 Hz, ^4^*J* = 1 Hz, 1H, H-5), 7.45–7.42 (m, 2H, H-12/H-13/H-14/H-15/H-16), 7.37–7.31 (m, 4H, H-4, H-12/H-13/H-14/H-15/H-16), 7.25 (d, ^3^*J* = 8 Hz, 1H, H-3), 6.72 (d, ^2^*J*_H′–H″_ = 15 Hz, 1H, H-8a/b), 5.64 (d, ^2^*J*_H′–H″_ = 15 Hz, 1H, H-11a/b), 5.48–5.42 (m, 2H, H-8a/b, H-11a/b), 3.91 (s, 3H, H-1), 1.50 (s, 15H, H-19). ^13^C{^1^H}-NMR (100.57 MHz, CDCl_3_); δ (ppm) 161.8 (C-18), 139.4 (C-9), 133.6 (C-17), 131.9 (C-2), 131.2 (C-7), 129.3 (C-12/C-13/C-14/C-15/C-16), 129.1 (C-10), 129.0 (C-12/C-13/C-14/C-15/C-16), 126.3 (C-5), 125.3 (C-4), 113.7 (C-6), 110.2 (C-3), 96.4 (d, ^1^J(Rh-C_Cp_) = 8 Hz, C-20), 55.5 (C-11), 39.1 (C-8), 33.0 (C-1), 8.8 (C-19).

*[Chlorido{1-[(1-benzyl-1,2,3-triazol-4-yl-κN)methyl]-3-methylbenzimidazole-2-thione-κS}(η^5^-pentamethylcyclopentadiene)iridium(III)] hexafluorophosphate***4a**, the synthesis of **4a** was performed according to general procedure A using **a** (50 mg, 0.150 mmol), [IrCl_2_(η^5^-pentamethylcyclopentadiene)]_2_ (59 mg, 0.075 mmol), and ammonium hexafluorophosphate (37 mg, 0.220 mmol) to afford a yellow powder (122 mg, 95%). Calcd. for C_28_H_32_ClF_6_IrN_5_PS·0.3H_2_O: C, 39.63; H, 3.87; N, 8.25%. Found: C, 39.26; H, 3.50; N, 8.14%. MS (ESI^+^): *m*/*z* = 698.1672 [M – PF_6_]^+^ (m_calc_ = 698.1696). ^1^H-NMR (399.89 MHz, CDCl_3_); δ (ppm) 8.54 (s, 1H, H-10), 7.77 (d, ^3^*J* = 8 Hz, 1H, H-3/H-6), 7.49 (dt, ^3^*J* = 7 Hz, ^4^*J* = 1 Hz, 1H, H-4/H-5), 7.44–7.36 (m, 6H, H-4/H-5, H-12, H-13, H-14, H-15, H-16), 7.34 (d, ^3^*J* = 8 Hz, 1H, H-3/H-6), 5.76 (d, ^2^*J*_H′–H″_ = 16 Hz, 1H, H-8a/b), 5.51 (s, 2H, H-11), 5.40 (d, ^2^*J*_H′–H″_ = 16 Hz, 1H, H-8a/b), 3.95 (s, 3H, H-1), 1.54 (s, 15H, H-19). ^13^C{^1^H}-NMR (100.57 MHz, CDCl_3_); δ (ppm) 161.9 (C-18), 138.2 (C-9), 132.7 (C-17), 132.0 (C-2), 130.8 (C-7), 129.4 (C-12/C-13/C-14/C-15/C-16), 129.3 (C-12/C-13/C-14/C-15/C-16), 129.1 (C-12/C-13/C-14/C-15/C-16), 126.5 (C-10), 126.4 (C-4/C-5), 125.7 (C-4/C-5), 111.1 (C-3/C-6), 110.8 (C-3/C-6), 89.4 (C-20), 55.9 (C-11), 38.4 (C-8), 33.1 (C-1), 8.5 (C-19).

*[Chlorido{1-[(1-benzyl-1,2,3-triazol-4-yl-κN)methyl]-3-methylbenzimidazole-2-thione-κS}(η^5^-pentamethylcyclopentadiene)iridium(III)] chloride***4a^Cl^**, the synthesis of **4a^Cl^** was performed according to general procedure B using **a** (50 mg, 0.150 mmol) and [IrCl_2_(η^5^-pentamethylcyclopentadiene)]_2_ (59 mg, 0.075 mmol) to afford a yellow powder (105 mg, 94%). Calcd. for C_28_H_32_Cl_2_IrN_5_S: C, 45.83; H, 4.40; N, 9.54%. Found: C, 45.47; H, 4.49; N, 9.94%. MS (ESI^+^): *m*/*z* = 662.1949 [M – 2Cl – H]^+^ (m_calc_ = 662.1929). ^1^H-NMR (399.89 MHz, CDCl_3_); δ (ppm) 9.98 (s, 1H, H-10), 8.81 (d, ^3^*J* = 8 Hz, 1H, H-6), 7.51 (t, ^3^*J* = 8 Hz, 1H, H-5), 7.43–7.32 (m, 6H, H-4, H-12, H-13, H-14, H-15, H-16), 7.29 (d, ^3^*J* = 8 Hz, 1H, H-3), 6.79 (d, ^2^*J*_H′–H″_ = 15 Hz, 1H, H-8a/b), 5.58 (d, ^2^*J*_H′–H″_ = 15 Hz, 1H, H-11a/b), 5.48 (d, ^2^*J*_H′–H″_ = 15 Hz, 1H, H-11a/b), 5.36 (d, ^2^*J*_H′–H″_ = 15 Hz, 1H, H-8a/b), 3.94 (s, 3H, H-1), 1.49 (s, 15H, H-19). ^13^C{^1^H}-NMR (100.57 MHz, CDCl_3_); δ (ppm) 161.5 (C-18), 138.4 (C-9), 133.3 (C-17), 131.8 (C-2), 131.3 (C-7), 129.2 (C-12/C-13/C-14/C-15/C-16), 129.1 (C-12/C-13/C-14/C-15/C-16), 128.6 (C-10), 126.5 (C-5), 125.4 (C-4), 113.8 (C-6), 110.2 (C-3), 89.0 (C-20), 55.7 (C-11), 39.2 (C-8), 33.2 (C-1), 8.4 (C-19).

*[Chlorido{1-[(1-benzyl-1,2,3-triazol-4-yl-κN)methyl]-3-methylimidazole-2-thione-κS}(η^6^-p-cymene)ruthenium(II)] hexafluorophosphate***1b**, the synthesis of **1b** was performed according to general procedure A using **b** (50 mg, 0.18 mmol), [RuCl_2_(η^6^-p-cymene)]_2_ (54 mg, 0.09 mmol), and potassium hexafluorophosphate (48 mg, 0.26 mmol) to afford a brownish yellow powder (112 mg, 91%). Calcd. for C_24_H_29_ClF_6_N_5_PRuS: C, 41.12; H, 4.17; N, 9.99%. Found: C, 41.48; H, 4.17; N, 9.63%. MS (ESI^+^): *m*/*z* = 556.0836 [M – PF_6_]^+^ (m_calc_ = 556.0876). ^1^H-NMR (399.89 MHz, CDCl_3_); δ (ppm) 8.24 (s, 1H, H-6), 7.45–7.34 (m, 5H, H-8, H-9, H-10, H-11, H-12), 7.23 (brs, 1H, H-3), 6.80 (brs, 1H, H-2), 5.59–5.47 (m, 3H, H-7a, H-7b, H-17/H-18/H-19/H-20), 5.40 (s, 2H, H-17/H-18/H-19/H-20, H-17/H-18/H-19/H-20), 5.35–5.28 (m, 2H, H-4a/b, H-17/H-18/H-19/H-20), 5.23 (d, ^2^*J*_H′–H″_ = 15 Hz, 1H, H-4a/b), 3.67 (s, 3H, H-1), 2.75 (sept, ^3^*J* = 7 Hz, 1H, H-22), 2.08 (s, 3H, H-15), 1.24 (d, ^4^*J* = 7 Hz, 3H, H-23/H-24), 1.18 (d, ^4^*J* = 7 Hz, 3H, H-23/H-24). ^13^C{^1^H}-NMR (100.57 MHz, CDCl_3_); δ (ppm) 154.8 (C-14), 139.6 (C-5), 132.9 (C-13), 129.4 (C-8/C-9/C-10/C-11/C-12), 128.8 (C-8/C-9/C-10/C-11/C-12), 126.9 (C-6), 121.4 (C-2/C-3), 120.5 (C-2/C-3), 104.9 (C-21), 98.9 (C-16), 84.9 (C-17/C-18/C-19/C-20), 84.7 (C-17/C-18/C-19/C-20), 84.3 (C-17/C-18/C-19/C-20), 83.8 (C-17/C-18/C-19/C-20), 55.8 (C-7), 41.3 (C-4), 36.5 (C-1), 30.6 (C-22), 22.2 (C-23/C-24), 22.0 (C-23/C-24), 18.3 (C-15).

*[Chlorido{1-[(1-benzyl-1,2,3-triazol-4-yl-κN)methyl]-3-methylimidazole-2-thione-κS}(η^6^-p-cymene)ruthenium(II)] chloride***1b^Cl^**, the synthesis of **1b^Cl^** was performed according to general procedure B using **b** (50 mg, 0.18 mmol) and [RuCl_2_(η^6^-*p*-cymene)]_2_ (54 mg, 0.09 mmol) to afford a brownish red powder (82 mg, 79%). Calcd. for C_24_H_29_Cl_2_N_5_RuS·0.25H_2_O: C, 48.36; H, 4.99; N, 11.75%. Found: C, 48.60; H, 5.12; N, 11.52%. MS (ESI^+^): *m*/*z* = 556.0871 [M – Cl]^+^ (m_calc_ = 556.0876). ^1^H-NMR (399.89 MHz, CDCl_3_); δ (ppm) 9.36 (brs, 1H, H-6), 8.19 (s, 1H, H-3), 7.43–7.34 (m, 5H, H-8, H-9, H-10, H-11, H-12), 6.74 (s, 1H, H-2), 6.43 (d, ^2^*J*_H′–H″_ = 15 Hz, 1H, H-4a/b), 5.63–5.53 (m, 2H, H-7), 5.49 (d, ^3^*J* = 6 Hz, 1H, H-17/H-18), 5.40 (d, ^3^*J* = 6 Hz, 1H, H-19/H-20), 5.35 (d, ^3^*J* = 6 Hz, 1H, H-17/H-18), 5.26 (d, ^3^*J* = 6 Hz, 1H, H-19/H-20), 5.19 (d, ^2^*J*_H′–H″_ = 15 Hz, 1H, H-4a/b), 3.65 (s, 3H, H-1), 2.65 (sept, ^3^*J* = 7 Hz, 1H, H-22), 2.02 (s, 3H, H-15), 1.20 (d, ^4^*J* = 7 Hz, 3H, H-23/H-24), 1.14 (d, ^4^*J* = 7 Hz, 3H, H-23/H-24). ^13^C{^1^H}-NMR (100.57 MHz, CDCl_3_); δ (ppm) 154.1 (C-14), 140.3 (C-5), 133.6 (C-13), 129.2 (C-8/C-9/C-10/C-11/C-12), 129.1 (C-6), 128.6 (C-8/C-9/C-10/C-11/C-12), 122.3 (C-3), 120.8 (C-2), 104.4 (C-21), 98.4 (C-16), 84.8 (C-17/C-18/C-19/C-20), 84.5 (C-17/C-18/C-19/C-20), 84.4 (C-17/C-18/C-19/C-20), 84.0 (C-17/C-18/C-19/C-20), 55.6 (C-7), 41.5 (C-4), 36.5 (C-1), 30.6 (C-22), 22.2 (C-23/C-24), 21.9 (C-23/C-24), 18.3 (C-15).

*[Chlorido{1-[(1-benzyl-1,2,3-triazol-4-yl-κN)methyl]-3-methylimidazole-2-thione-κS}(η^6^-p-cymene)osmium(II)] hexafluorophosphate***2b**, the synthesis of **2b** was performed according to general procedure B using **b** (50 mg, 0.18 mmol), [OsCl_2_(η^6^-p-cymene)]_2_ (69 mg, 0.09 mmol), and potassium hexafluorophosphate (48 mg, 0.26 mmol) to afford a yellow powder (125 mg, 90%). Calcd. for C_24_H_29_ClF_6_N_5_OsPS 0.3C_6_H_14_: C, 37.97; H, 4.10; N, 8.58%. Found: C, 38.07; H, 4.12; N, 8.60%. MS (ESI^+^): *m*/*z* = 646.1422 [M–PF_6_]^+^ (m_calc_ = 646.1447). ^1^H-NMR (399.89 MHz, CDCl_3_); δ (ppm) 8.29 (s, 1H, H-6), 7.43–7.34 (m, 5H, H-8/H-9/H-10/H-11/H-12), 7.32 (d, ^3^*J* = 2 Hz, 1H, H-3), 6.84 (d, ^3^*J* = 2 Hz, 1H, H-2), 5.79 (d, ^3^*J* = 6 Hz, 1H, H-17/H-18), 5.70 (d, ^3^*J* = 6 Hz, 1H, H-17/H-18), 5.64 (d, ^3^*J* = 6 Hz, 1H, H-19/H-20), 5.59 (d, ^3^*J* = 6 Hz, 1H, H-19/H-20), 5.55 (d, ^2^*J*_H′–H″_ = 14 Hz, 1H, H-7a/b), 5.49 (d, ^2^*J*_H′–H″_ = 14 Hz, 1H, H-7a/b), 5.42 (d, ^2^*J*_H′–H″_ = 15 Hz, 1H, H-4a/b), 5.27 (d, ^2^*J*_H′–H″_ = 15 Hz, 1H, H-4a/b), 3.69 (s, 3H, H-1), 2.64 (sept, ^3^*J* = 7 Hz, 1H, H-22), 2.11 (s, 3H, H-15), 1.22 (d, ^4^*J* = 7 Hz, 3H, H-23/H-24), 1.19 (d, ^4^*J* = 7 Hz, 3H, H-23/H-24). ^13^C{^1^H}-NMR (100.57 MHz, CDCl_3_); δ (ppm) 156.3 (C-14), 138.0 (C-5), 132.7 (C-13), 129.4 (C-8/C-9/C-10/C-11/C-12), 129.3 (C-8/C-9/C-10/C-11/C-12), 128.7 (C-8/C-9/C-10/C-11/C-12), 126.5 (C-6), 121.2 (C-2/C-3), 120.7 (C-2/C-3), 95.8 (C-21), 90.9 (C-16), 76.1 (C-17/C-18/C-19/C-20), 76.0 (C-17/C-18/C-19/C-20), 55.7 (C-7), 41.3 (C-4), 36.7 (C-1), 30.7 (C-22), 22.5 (C-23/C-24), 22.1 (C-23/C-24), 18.2 (C-15).

*[Chlorido{1-[(1-benzyl-1,2,3-triazol-4-yl-κN)methyl]-3-methylimidazole-2-thione-κS}(η^6^-p-cymene)osmium(II)] chloride***2b^Cl^**, the synthesis of **2b^Cl^** was performed according to general procedure B using **b** (50 mg, 0.18 mmol) and [OsCl_2_(η^6^-*p*-cymene)]_2_ (69 mg, 0.09 mmol) to afford a yellow powder (71 mg, 60%). MS (ESI^+^): *m*/*z* = 646.1422 [M–Cl]^+^ (m_calc_ = 646.1447). ^1^H-NMR (399.89 MHz, CDCl_3_); δ (ppm) 9.38 (s, 1H, H-6), 8.31 (d, ^3^*J* = 2 Hz, 1H, H-3), 7.42–7.32 (m, 5H, H-8/H-9/H-10/H-11/H-12), 6.77 (d, ^3^*J* = 2 Hz, 1H, H-2), 6.59 (d, ^2^*J*_H′–H″_ = 14 Hz, 1H, H-4a/b), 5.75 (d, ^3^*J* = 6 Hz, 1H, H-17/H-18), 5.63 (s, 2H, H-17/H-18/H-19/H-20), 5.59 (d, ^2^*J*_H′–H″_ = 14 Hz, 1H, H-7a/b), 5.53 (d, ^2^*J*_H′–H″_ = 14 Hz, 1H, H-7a/b), 5.51 (d, ^3^*J* = 6 Hz, 1H, H-19/H-20), 5.19 (d, ^2^*J*_H′–H″_ = 14 Hz, 1H, H-4a/b), 3.68 (s, 3H, H-1), 2.55 (sept, ^3^*J* = 7 Hz, 1H, H-22), 2.05 (s, 3H, H-15), 1.19 (d, ^4^*J* = 7 Hz, 3H, H-23/H-24), 1.16 (d, ^4^*J* = 7 Hz, 3H, H-23/H-24). ^13^C{^1^H}-NMR (100.57 MHz, CDCl_3_); δ (ppm) 155.6 (C-14), 138.7 (C-5), 133.4 (C-13), 129.2 (C-8/C-9/C-10/C-11/C-12), 128.5 (C-8/C-9/C-10/C-11/C-12), 128.2 (C-6), 122.3 (C-3), 120.5 (C-2), 95.2 (C-21), 90.6 (C-16), 76.6 (C-17/C-18/C-19/C-20), 76.2 (C-17/C-18/C-19/C-20), 76.0 (C-17/C-18/C-19/C-20), 75.9 (C-17/C-18/C-19/C-20), 55.6 (C-7), 41.0 (C-4), 36.6 (C-1), 30.7 (C-22), 22.7 (C-23/C-24), 22.0 (C-23/C-24), 18.2 (C-15).

*[Chlorido{1-[(1-benzyl-1,2,3-triazol-4-yl-κN)methyl]-3-methylimidazole-2-thione-κS}(η^5^-pentamethylcyclopentadiene)rhodium(III)] hexafluorophosphate***3b**, the synthesis of **3b** was performed according to general procedure A using **b** (53 mg, 0.19 mmol), [RhCl_2_(η^5^-pentamethylcyclopentadiene)]_2_ (57 mg, 0.09 mmol), and potassium hexafluorophosphate (51 mg, 0.28 mmol) to afford an orange powder (128 mg, 72%). Calcd. for C_24_H_30_ClF_6_N_5_PRhS: C, 40.95; H, 4.30; N, 9.95%. Found: C, 41.24; H, 4.01; N, 9.71%. MS (ESI^+^): *m*/*z* = 524.1357 [M – Cl – PF_6_ – 3H + 2D]^+^ (m_calc_ = 524.1324). ^1^H-NMR (399.89 MHz, CDCl_3_); δ (ppm) 8.3 (s, 1H, H-6), 7.43–7.34 (m, 5H, H-8, H-9, H-10, H-11, H-12), 7.22 (d, ^3^*J* = 3 Hz, 1H, H-3), 6.82 (d, ^3^*J* = 3 Hz, 1H, H-2), 5.54 (d, ^2^*J*_H′–H″_ = 14 Hz, 1H, H-7a/b), 5.48 (d, ^2^*J*_H′–H″_ = 14 Hz, 1H, H-7a/b), 5.38 (d, ^2^*J*_H′–H″_ = 15 Hz, 1H, H-4a/b), 5.28 (d, ^2^*J*_H′–H″_ = 15 Hz, 1H, H-4a/b), 3.71 (s, 3H, H-1), 1.56 (s, 15H, H-15). ^13^C{^1^H}-NMR (100.57 MHz, CDCl_3_); δ (ppm) 154.2 (C-14), 139.6 (C-5), 132.9 (C-13), 129.3 (C-8/C-9/C-10/C-11/C-12), 128.9 (C-8/C-9/C-10/C-11/C-12), 126.5 (C-6), 121.2 (C-2), 120.2 (C-3), 96.4 (d, ^1^J(Rh-C_Cp_) = 7 Hz, C-16), 55.7 (C-7), 41.3 (C-4), 36.5 (C-1), 8.9 (C-15).

*[Chlorido{1-[(1-benzyl-1,2,3-triazol-4-yl-κN)methyl]-3-methylimidazole-2-thione-κS}(η^5^-pentamethylcyclopentadiene)rhodium(III)] chloride***3b^Cl^**, the synthesis of **3b^Cl^** was performed according to general procedure B using **b** (53 mg, 0.19 mmol) and [RhCl_2_(η^5^-pentamethylcyclopentadiene)]_2_ (57 mg, 0.09 mmol) to afford an orange-red powder (100 mg, 95%). MS (ESI^+^): *m*/*z* = 524.1338 [M–2Cl–3H + 2D]^+^ (m_calc_ = 524.1324). ^1^H-NMR (399.89 MHz, CDCl_3_); δ (ppm) 9.37 (s, 1H, H-6), 8.20 (d, ^3^*J* = 2 Hz, 1H, H-3), 7.39–7.34 (m, 5H, H-8/H-9/H-10/H-11/H-12), 6.76 (d, ^3^*J* = 2 Hz, 1H, H-2), 6.50 (d, ^2^*J*_H′–H″_ = 14 Hz, 1H, H-4a/b), 5.57 (d, ^2^*J*_H′–H″_ = 14 Hz, 1H, H-7a/b), 5.52 (d, ^2^*J*_H′–H″_ = 14 Hz, 1H, H-7a/b), 5.19 (d, ^2^*J*_H′–H″_ = 14 Hz, 1H, H-4a/b), 3.70 (s, 3H, H-1), 1.71 (s, 15H, H-15). ^13^C{^1^H}-NMR (100.57 MHz, CDCl_3_); δ (ppm) 153.2 (C-14), 140.4 (C-5), 133.6 (C-13), 129.2 (C-8/C-9/C-10/C-11/C-12), 128.7 (C-8/C-9/C-10/C-11/C-12), 128.2 (C-6), 121.8 (C-3), 120.6 (C-2), 96.1 (d, ^1^J(Rh-C_Cp_) = 7 Hz, C-16), 55.6 (C-7), 41.0 (C-4), 36.4 (C-1), 8.9 (C-15).

*[Chlorido{1-[(1-benzyl-1,2,3-triazol-4-yl-κN)methyl]-3-methylimidazole-2-thione-κS}(η^5^-pentamethylcyclopentadiene)iridium(III)] hexafluorophosphate***4b**, the synthesis of **4b** was performed according to general procedure A using **b** (51 mg, 0.18 mmol), [IrCl_2_(η^5^-pentamethylcyclopentadiene)]_2_ (71 mg, 0.09 mmol), and potassium hexafluorophosphate (49 mg, 0.27 mmol) to afford a yellow powder (132 mg, 94%). Calcd. for C_24_H_30_ClF_6_IrN_5_PS 0.05C_6_H_14_: C, 36.60; H, 3.88; N, 8.78%. Found: C, 36.85; H, 3.48; N, 8.43%. MS (ESI^+^): *m*/*z* = 612.1848 [M – PF_6_ – Cl – H]^+^ (m_calc_ = 612.1769). ^1^H-NMR (399.89 MHz, CDCl_3_); δ (ppm) 8.39 (s, 1H, H-6), 7.43–7.34 (m, 5H, H-8, H-9, H10, H-11, H-12), 7.32 (d, ^3^*J* = 3 Hz, 1H, H-3), 6.86 (d, ^3^*J* = 3 Hz, 1H, H-2), 5.56–5.46 (m, 3H, H-4a/b, H-7), 5.21 (d, ^2^*J*_H′–H″_ = 15 Hz, 1H, H-4a/b, 3.75 (s, 3H, H-1), 1.53 (s, 15H, H-15). ^13^C{^1^H}-NMR (100.57 MHz, CDCl_3_); δ (ppm) 153.6 (C-14), 138.5 (C-5), 132.7 (C-13), 129.4 (C-8/C-9/C-10/C-11/C-12), 129.3 (C-8/C-9/C-10/C-11/C-12), 129.0 (C-8/C-9/C-10/C-11/C-12), 126.3 (C-6), 121.1 (C-2), 120.5 (C-3), 89.0 (C-16), 55.8 (C-7), 41.3 (C-4), 36.7 (C-1), 8.5 (C-15).

*[Chlorido{1-[(1-benzyl-1,2,3-triazol-4-yl-κN)methyl]-3-methylimidazole-2-thione-κS}(η^5^-pentamethylcyclopentadiene)iridium(III)] chloride***4b^Cl^**, the synthesis of **4b^Cl^** was performed according to general procedure B using **b** (51 mg, 0.18 mmol) and [IrCl_2_(η^5^-pentamethylcyclopentadiene)]_2_ (71 mg, 0.09 mmol) to afford a yellow powder (83 mg, 68%). Calcd. for C_24_H_30_Cl_2_IrN_5_S·0.55CH_4_O·0.05H_2_O: C, 41.99; H, 4.64; N, 9.97%. Found: C, 41.92; H, 4.56; N, 9.89%. MS (ESI^+^): *m*/*z* = 612.1778 [M – 2Cl – H]^+^ (m_calc_ = 612.1773). ^1^H-NMR (399.89 MHz, CDCl_3_); δ (ppm) 9.43 (s, 1H, H-6), 8.31 (d, ^3^*J* = 2 Hz, 1H, H-3), 7.38–7.35 (m, 5H, H-8/H-9/H-10/H-11/H-12), 6.79 (d, ^3^*J* = 2 Hz, 1H, H-2), 6.57 (d, ^2^*J*_H′–H″_ = 15 Hz, 1H, H-4a/b), 5.57 (d, ^2^*J*_H′–H″_ = 15 Hz, 1H, H-7a/b), 5.52 (d, ^2^*J*_H′–H″_ = 15 Hz, 1H, H-7a/b), 5.12 (d, ^2^*J*_H′–H″_ = 15 Hz, 1H, H-4a/b), 3.73 (s, 3H, H-1), 1.74 (s, 15H, H-15). ^13^C{^1^H}-NMR (100.57 MHz, CDCl_3_); δ (ppm) 152.7 (C-14), 139.4 (C-5), 133.3 (C-13), 129.2 (C-8/C-9/C-10/C-11/C-12), 128.8 (C-8/C-9/C-10/C-11/C-12), 127.9 (C-6), 121.9 (C-3), 120.5 (C-2), 88.7 (C-16), 55.6 (C-7), 41.0 (C-4), 36.6 (C-1), 8.4 (C-15).

### 3.3. Sulforhodamine B Cytotoxicity Assay

HCT116, SW480, and NCI-H460 cells were supplied by ATCC (Manassas, VA, USA), while SiHa cells were from Dr David Cowan, Ontario Cancer Institute, Canada. The cells were grown in αMEM (ThermoFisher Scientific, Auckland, New Zealand) supplemented with 5% fetal calf serum (Moregate Biotech, Hamilton, New Zealand) at 37 °C in a humidified incubator with 5% CO_2_. They were seeded at 750 (HCT116, NCI-H460), 4000 (SiHa) or 5000 (SW480) cells/well in 96-well plates and left to settle for 24 h. The compounds were added to the plates in a series of 3-fold dilutions, containing a maximum of 0.5% DMSO at the highest concentration. The assay was terminated after 72 h by addition of 10% trichloroacetic acid (Merck Millipore, Auckland, New Zealand) at 4 °C for 1 h. The cells were stained with 0.4% sulforhodamine B (Sigma-Aldrich, Auckland, New Zealand) in 1% acetic acid for 30 min in the dark at room temperature and then washed with 1% acetic acid to remove unbound dye. The stain was dissolved in unbuffered Tris base (10 mM; Serva, Heidelberg, Germany) for 30 min on a plate shaker in the dark and quantified on a BioTek EL808 micro-plate reader at an absorbance wavelength of 490 nm with 450 nm as the reference wavelength to determine the percentage of cell growth inhibition by determining the absorbance of each sample relative to a negative (no inhibitor) and a no-growth control (day 0). The IC_50_ values were calculated with SigmaPlot 12.5 using a three-parameter logistic sigmoidal dose−response curve between the calculated growth inhibition and the compound concentration. The presented IC_50_ values are the mean of at least 3 independent experiments, where 10 concentrations were tested in duplicate for each compound.

### 3.4. X-ray Fluorescence Microscopy (XFM)

#### 3.4.1. Sample Preparation

Silicon nitride membranes (Silson Ltd., Warwickshire, England) were washed for 2 min each in Milli-Q water, 70% ethanol, and 100% ethanol in a small petri dish. The membranes were air dried under sterile conditions and transferred into wells of 12-well culture plates which were exposed to UV-light overnight.

SKOV-3 cells were kindly provided by Dr Carmela Ricciardelli from the Robinson Research Institute at The University of Adelaide, Australia. The cells were cultured in a T75 flask and collected after trypsinizing (0.25% trypsin and ethylenediaminetetraacetic acid) for 3 min. The cells were spun down at 1200 rpm for 5 min. Cell supernatant was discarded and pellet suspended in 1 mL culturing media (SKOV-3: DMEM/F12, 10% FCS, l-Glutamine, 1% penicillin/streptomycin and 0.1% fungizone). Cell suspensions were mixed with trypan blue solution (0.4%) and transferred into a cell counting chamber slide (Thermo Fisher Scientific Australia, Adelaide, Australia). The viable cells were counted with Countess II (Thermo Fisher Scientific Australia, Adelaide, Australia). The cell solutions were prepared with culturing media and 2000 SKOV-3 cells were seeded onto each membrane without making contact with the pipette tip. The cells were incubated for 3 h at 37 °C and 5% CO_2_ atmosphere for attachment. Culturing media (1.4 mL) was added carefully to each well and the cells were incubated overnight at 37 °C, 5% CO_2_.

#### 3.4.2. Metal Complex Incubation

Complex **4a** (20 µM, 1% DMSO in media) was added to the wells carefully in 2 separate portions of 0.7 mL, enough to soak the cell-coated silicon nitride membrane in solution. The cells were incubated for 4 h. The compound solution was aspirated from the well carefully, avoiding contact with the membrane, and then the membrane was washed with PBS (0.7 mL) and aspirated. The cells were fixed onto the membrane with 4% buffered paraformaldehyde (0.7 mL). After 5 min, the paraformaldehyde solution was aspirated. The membrane was washed twice with PBS (0.7 mL) and aspirated. A solution of ammonium acetate (100 mM, 0.7 mL) was added to the membrane carefully and soaked for 2 min, after which ammonium acetate was aspirated. The latter step was repeated before Milli-Q water (0.7 mL) was added to cover the surface of the membrane and to wash out excess ammonium acetate. Milli-Q water was removed immediately by blotting with delicate tissue and the membrane was then allowed to dry completely while protected from dust. The membrane was stored at room temperature for subsequent experimentation.

#### 3.4.3. Advanced Photon Source and Operating Conditions

The distributions of P, Zn, and Ir in SKOV-3 cells incubated with **4a** were mapped at the 2-ID-D beamline at the Advanced Photon Source (Argonne National Laboratory, Lemont, IL) with modifications to the protocol described previously [55,56,57]. The beamline used a double multilayer monochromator and a gold “high flux” zone plate setup to focus a monochromatic beam into a spot of 300–400 nm full width at half maximum (FWHM) in diameter. Cells treated with **4a** were imaged with an incident X-ray energy of 13.1 keV to excite at the L-edge of Ir; the P and Zn maps for these cells were generated from K-edge fluorescence of these elements. The same Fresnel zone plate arrangement was used to focus the X-ray beam to the same spot size at the lower energy. An energy-dispersive silicon drift detector (Vortex EM, SII Nanotechnology, Northridge, CA, USA) was used to collect the X-ray fluorescence spectra from the sample, which was placed in a He environment at an angle of 75° to the incident beam. All elemental maps were recorded in fly-scan mode, with a 0.5 μm step-size in x and y direction, a 150 ms dwell time for the **4a**-treated cell maps. Elemental maps were generated with the MAPS software package [58] by Gaussian fitting of the raw emission spectra for each image pixel. The Gaussian peaks were matched to characteristic X-ray emission lines to determine the fluorescence signal for each element. Quantification of the data (in μg/cm^2^) was performed by comparing the X-ray fluorescence intensity to those from National Bureau of Standards thin film standards NBS-1832, NBS-1833 (National Bureau of Standards, Gaithersburg, MD, USA).

### 3.5. DFT Calculations

GAUSSIAN 09W [59] was used to calculate the optimized ground state structures and frequencies for the different molecules by density functional theory (DFT) with the B3LYP-D3 hybrid exchange functional and a split basis set for C, H, N, S, and Cl (6-31G(d,p)) and the transition metals iridium, osmium, rhodium, and ruthenium (SDDAll) in vacuum. The SCRF (self-consistent reaction field) keyword was implemented for the optimization of the molecules solvated in chloroform. This method is the integral equation formalism variant of the Polarizable Continuum Model (IEFPCM) [60]. The EmpiricalDispersion = GD3 keyword was implemented for the empirical dispersion correction for the optimization of the molecules [61]. The NMR keyword was employed for the calculation of ^1^H-NMR spectra [62,63,64,65,66,67].

### 3.6. Stability Studies

For the stability studies in DMSO, **1a**, **1a^Cl^**, **4a**, and **4a^Cl^** (1–2 mg) were dissolved in DMSO-*d*_6_ and ^1^H-NMR spectra were recorded over a period of 48 h. To determine the stability in aqueous solution, **1b**, **2b**, **3b**, and **4b** (1–2 mg) were dissolved in DMSO-*d*_6_ and immediately diluted with D_2_O to reach a DMSO content of 15vol% and ^1^H-NMR spectra were recorded over a period of 120 h.

## 4. Conclusions

A series of [M^II/III^(cym/Cp*)Cl]X complexes featuring benzimidazole- or imidazole-thione ligands was synthesized as cationic salts with X = Cl^−^ as the counterion, and they were converted also into their X = PF_6_^−^ derivatives. In the latter step, addition of NH_4_PF_6_ caused unexpected Cl^−^/NH_3_ ligand exchange at the metal center, as was determined crystallographically and by ESI-MS. To circumvent this reaction, KPF_6_ was used instead of NH_4_PF_6_ which gave rise of pure products. All the prepared complexes were characterized by 1D and 2D NMR spectroscopy, elemental analysis, and/or ESI-MS. Single-crystal X-ray diffraction analysis of complexes with different counterions revealed hydrogen bonding C–H···X between some of the protons sitting in a pocket formed from the imidazole/benzimidazole, methylene, and triazole moieties with the counterions. This confirmed ^1^H-NMR spectroscopic investigations on complexes with different counterions (Cl^−^ vs. PF_6_^−^ vs. BF_4_^−^) in which several protons shifted significantly in dependence of the counterion, with the protons in complexes with Cl^−^ counterions resonating at lowest field and with PF_6_^−^ at highest field. The ^1^H-NMR spectra obtained from DFT calculations supported the experimental observations. The complexes showed good stability in aqueous solution. The in vitro cytotoxicity studies showed that the Ir benzimidazole derivative **4a** was the most potent antiproliferative agent in all four cell lines with IC_50_ values of around 20 μM, indicating moderate activity. The lack of cytotoxic activity of the imidazole-derived complexes suggests that lipophilicity may be a key factor for this class of complexes to achieve a higher degree of cytotoxic activity. The other factor influencing the cytotoxicity was the nature of the metal center with less kinetically labile Os and Ir derivatives being more potent than their Ru and Rh counterparts. Moreover, XFM revealed that there was a significant amount of cell cytoplasm uptake of **4a** into SKOV-3 cells, which suggests that the mode of action of this complex type is unlikely to be DNA dependent.

## Figures and Tables

**Table 1:** Key bond lengths (Å) and angles (°) for **2a^Cl^**, **3b**, **3b^Cl^**, **4a**, **4b^Cl^**, and **1b^NH3^**.

Complex	2a^Cl^	3b	3b^Cl^	4a	4b^Cl^	1b^NH3^
	Bond length/Å					
M–X *^a^*	2.4110(5)	2.424(1)	2.4137(8)	2.4122(8)	2.409(1)	2.152(2)
M–S	2.4147(6)	2.426(1)	2.4276(8)	2.4091(8)	2.396(1)	2.3895(4)
M–N_tri_	2.127(2)	2.139(5)	2.112(3)	2.089(3)	2.090(4)	2.110(2)
C=S	1.698(3)	1.713(6)	1.699(3)	1.706(3)	1.716(5)	1.717(2)
M–Cp*_centroid_	1.666	1.791	1.786	1.790	1.785	1.679
	Bond angle/°					
S–M–N_tri_	96.90(6)	96.4(1)	97.14(7)	97.60(8)	96.7(1)	95.14(4)
S–M–X *^a^*	86.75(2)	88.79(5)	92.22(3)	88.49(3)	89.90(4)	86.66(5)
N_tri_–M–X *^a^*	83.77(5)	85.6(1)	87.33(7)	86.28(8)	86.0(1)	81.69(6)
M–S=C	113.00(9)	107.3(2)	111.6(1)	115.1(1)	112.6(2)	114.12(6)

*^a^* X = Cl for **2a^Cl^**, **3b**, **3b^Cl^**, **4a**, and **4b^Cl^**, and X = N_NH3_ for **1b^NH3^**.

**Table 2:** Simulated (δ_sim_) and experimental (δ_exp_) ^1^H-NMR shifts of H-3, H-4b and H-6 in **3b**, **3b^BF4^**, and **3b^Cl^**.

Complex	Proton	δ_exp_/ppm	δ_sim_/ppm	Δ(δ_exp_ − δ_sim_)/ppm
**3b**	H-3	7.22	7.05	0.17
	H-4b	5.38	5.04	0.34
	H-6	8.30	8.01	0.29
**3b^BF4^**	H-3	7.47	6.95	0.52
	H-4b	5.58	5.26	0.32
	H-6	8.58	9.02	0.44
**3b^Cl^**	H-3	7.85	7.92	0.07
	H-4b	6.08	6.14	0.06
	H-6	9.20	9.06	0.14

**Table 3:** Antiproliferative IC_50_ values (µM) for complexes **1a**–**4b** against HCT116 (human colorectal carcinoma), NCI-H460 (human non-small cell lung carcinoma), SiHa (human cervical carcinoma), and SW480 (human colon adenocarcinoma) cancer cells expressed as mean ± standard error (n = 3).

Compound	IC_50_ Values (μM)			
	HCT116	NCI-H460	SiHa	SW480
**1a**	61 ± 27	>100	>100	>100
**2a**	42 ± 6	45 ± 6	>100	>100
**3a**	76 ± 39	>100	>100	>100
**4a**	20 ± 3	25 ± 1	71 ± 4	83 ± 24
**1b**	>100	>100	>100	>100
**2b**	>100	>100	>100	>100
**3b**	>100	>100	>100	>100
**4b**	>100	>100	>100	>100

## Supplementary Materials

The following are available online. ESI-MS data, ^1^H-NMR spectra, molecular structures, and data collection parameters for single crystal X-ray diffraction analyses, calculated ^1^H-NMR spectra, stability studies in DMSO-*d*_6_ and 15% DMSO-*d*_6_/D_2_O, and ^1^H, ^13^C{^1^H}-NMR and ESI-mass spectra.
