# Supplementary material for: Thiourea-Derived Chelating Ligands and Their Organometallic Compounds: Investigations into Their Anticancer Activity
Source: Molecules. 2020 Aug 11;25(16):3661. doi: 10.3390/molecules25163661 (PMC7464268; doi:10.3390/molecules25163661)
Supplement: Supplementary file 1 [file molecules-25-03661-s001.pdf]

molecules

Article

Thiourea-Derived Chelating Ligands and Their Organometallic Compounds: Investigations into Their Anticancer Inhibitory Activity

Kelvin K.H. Tong [1], Muhammad Hanif [1], James H. Lovett [2], Katja Hummitzsch [3], Hugh H. Harris [2], Tilo Söhnel [1], Stephen M. F. Jamieson [4] and Christian G. Hartinger [1,*]

[1] School of Chemical Sciences, University of Auckland, Private Bag 92019, Auckland 1142, New Zealand
[2] Department of Chemistry, The University of Adelaide, Adelaide, 5005 SA, Australia
[3] Discipline of Obstetrics and Gynecology, The University of Adelaide, Robinson Research Institute, Adelaide, 5005 SA, Australia
[4] Auckland Cancer Society Research Centre, University of Auckland, Private Bag 92019, Auckland 1142, New Zealand
* Correspondence: c.hartinger@auckland.ac.nz; Tel.: +64-9-3737599 83220 (C.G.H.); web: http://hartinger.auckland.ac.nz/

Received: date; Accepted: date; Published: date

Table of Contents

ESI-MS data
^{1}H NMR spectra
Molecular structures and data collection parameters for XRD analyses
Calculated ^{1}H NMR spectra
Stability studies in DMSO-*d*$_6$ and 15% DMSO-*d*$_6$/D_2O
^{1}H, ^{13}C{^{1}H} NMR and ESI-mass spectra

Table S1. ESI-MS data for compounds **a**, **b**, **1a**-**4b**, **1a**^{Cl}-**4b**^{Cl} and **1b**^{NH3} analyzed in positive ion mode.

Compound	Observed *m*/*z*	Theoretical *m*/*z*	Assignment
**a**	358.1106	358.1102	$[M + Na]^+$
**b**	308.0942	308.0946	$[M + Na]^+$
**1a**	606.1034	606.1032	$[M - PF_6]^+$
**1a**^{Cl}	606.1024	606.1032	$[M - Cl]^+$
**2a**	696.1576	696.1603	$[M - PF_6]^+$
**2a**^{Cl}	696.1576	696.1603	$[M - Cl]^+$
**3a**	608.1125	608.1122	$[M - PF_6]^+$
**3a**^{Cl}	574.1487	574.1506	$[M - 2Cl - H]^+$
**4a**	698.1672	698.1696	$[M - PF_6]^+$
**4a**^{Cl}	662.1949	662.1929	$[M - 2Cl - H]^+$
**1b**	556.0836	556.0876	$[M - PF_6]^+$
**1b**^{Cl}	556.0871	556.0876	$[M - Cl]^+$
**1b**^{NH3}	683.1120	683.1094	$[M - PF_6]^+$
**2b**	646.1422	646.1447	$[M - PF_6]^+$
**2b**^{Cl}	646.1422	646.1447	$[M - Cl]^+$
**3b**	524.1357	524.1324	$[M - Cl - PF_6 - 3H + 2D]^+$
**3b**^{Cl}	524.1338	524.1324	$[M - 2Cl - 3H + 2D]^+$
**4b**	612.1848	612.1769	$[M - Cl - PF_6 - H]^+$
**4b**^{Cl}	612.1778	612.1773	$[M - 2Cl - H]^+$

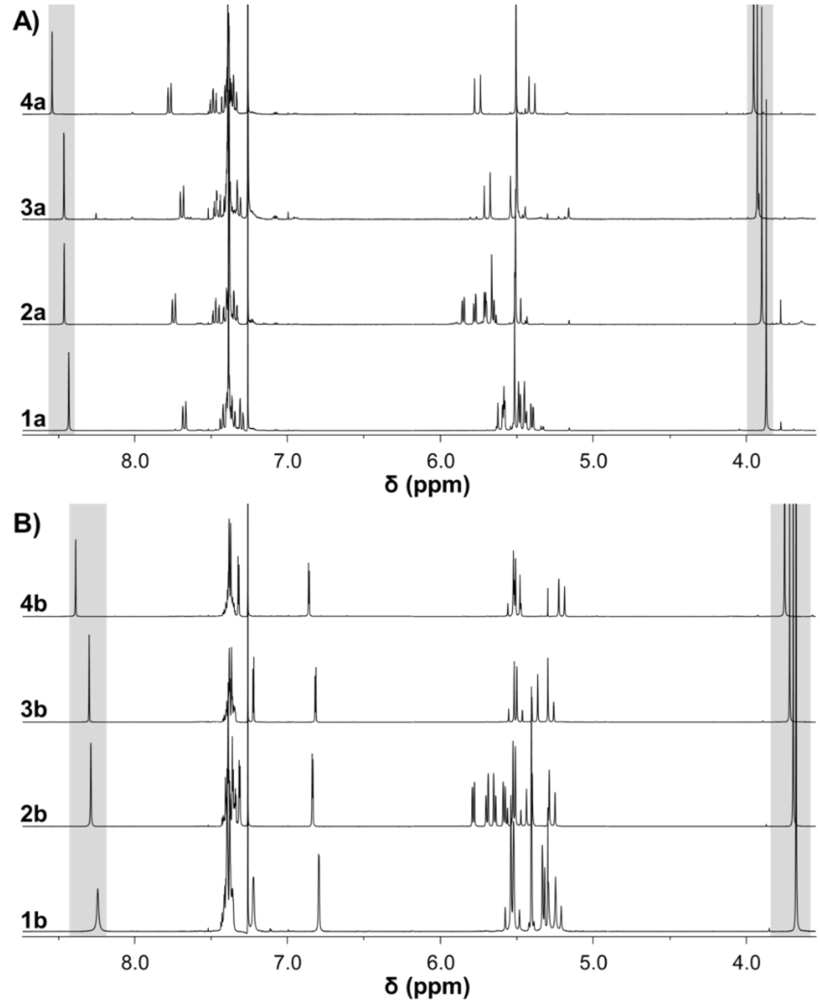

Figure S1. Comparison of the ^{1}H NMR spectra of complexes **1a**-**4a** (A) and **1b**–**4b** (B). The triazole and methyl H peaks shifting in dependence of the metal center are highlighted with grey boxes.

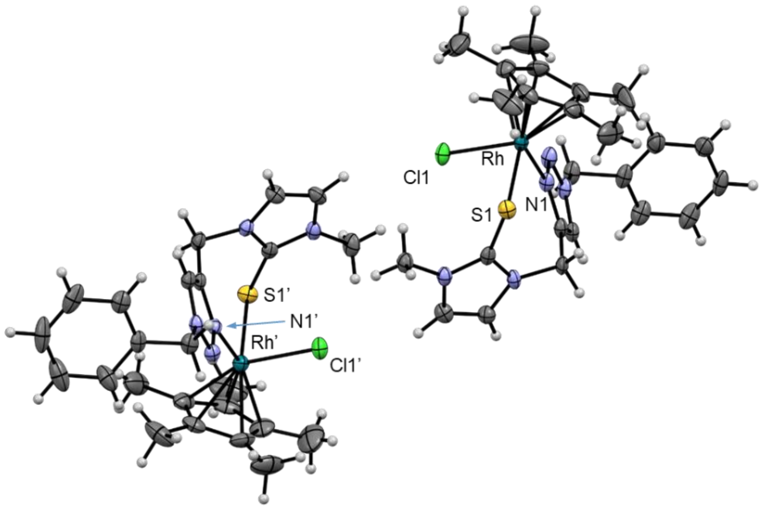

Figure S2. Molecular structures of the two enantiomers of **3b**. Counterions have been omitted for clarity and the structures are shown at 50% probability level.

molecules

MDPI

Table S2. X-ray crystallographic data for complex **1b**^{NH3}, **2a**, **2a**^{Cl}, **3b**, **3b**^{Cl}, **4a**, and **4b**^{Cl}.

	**1b**^{NH3}	**2a**$^{Cl}\cdot H_2O$	**3b**·toluene	**3b**$^{Cl}\cdot H_2O$·toluene	**4a**·$CHCl_3$	**4b**$^{Cl}\cdot H_2O$
**CCDC**	2013780	2013781	2013782	2013783	2013784	2013785
**Formula**	$C_{24}H_{32}N_6RuSP_2F_{12}$	$C_{28}H_{31}Cl_2N_5OsS\cdot$ 2.25H_2O	$C_{24}H_{30}ClN_5RhSPF_6\cdot$ 0.5C_7H_8 [+H_2O]#	$C_{24}H_{30}Cl_2N_5RhS\cdot$ 1.5H_2O [+ C_7H_8 + H_2O]#	$C_{28}H_{32}ClIrN_5SPF_6\cdot$ 3$CHCl_3$	$C_{24}H_{30}Cl_2IrN_5S\cdot$ 1.25H_2O
**Formula weight [g mol^{-1}]**	827.62	771.27	749.98	621.42	1201.37	706.21
**Temperature [K]**	99.8(3)	100.0(1)	100.0(2)	100.0(1)	100.0(1)	100.0(1)
**Crystal System**	monoclinic	monoclinic	orthorhombic	triclinic	monoclinic	triclinic
**Space group**	*P*2$_1$/*n*	*P*2$_1$/*n*	*Pna*2$_1$	*P*-1	*P*2$_1$/*c*	*P*-1
***a* [Å]**	9.06730(9)	10.77920(10)	29.9478(3)	8.49050(10)	12.49577(7)	8.3119(2)
***b* [Å]**	12.95883(13)	9.14240(10)	8.43080(10)	18.6371(2)	11.25335(6)	18.9967(3)
***c* [Å]**	27.5090(2)	29.8464(3)	25.6410(3)	19.1728(2)	32.00737(18)	19.2510(3)
**α [º]**	90	90	90	96.503(1)	90	62.526(1)
**β [º]**	97.9021(9)	90.727(1)	90	90.247(1)	94.3484(5)	85.408(1)
**γ [º]**	90	90	90	94.121(1)	90	80.034(1)
**V [Å^3]**	3201.66(5)	2941.06(5)	6473.94(13)	3006.32(6)	4487.90(4)	2656.20(9)
**Z**	4	4	8	4	4	4
**ρ_{calc} [g/cm^3]**	1.717	1.742	1.539	1.478	1.778	1.766
**μ [mm^{-1}]**	6.424	10.818	6.640	7.124	12.506	12.540
**F(000)**	1664.0	1530.0	3048.0	1378.0	2352.0	1394.0
**Crystal size [mm^3]**	0.10 × 0.10 × 0.05	0.08 × 0.08 × 0.01	0.05 × 0.05 × 0.01	0.20 × 0.12 × 0.08	0.12 × 0.10 × 0.05	0.50 × 0.01 × 0.01
**2Θ range for data collection [°]**	11.898 to 135.472	11.35 to 135.454	11.436 to 136.492	11.174 to 135.476	11.086 to 136.496	11.362 to 136.482
**Index ranges**	-10 ≤ h ≤ 10, -15 ≤ k ≤ 15, -28 ≤ l ≤ 32	-12 ≤ h ≤ 12, -10 ≤ k ≤ 10, -35 ≤ l ≤ 35	-36 ≤ h ≤ 33, -7 ≤ k ≤ 10, -30 ≤ l ≤ 30	-10 ≤ h ≤ 10, -22 ≤ k ≤ 20, -23 ≤ l ≤ 23	-15 ≤ h ≤ 14, -13 ≤ k ≤ 13, -38 ≤ l ≤ 38	-10 ≤ h ≤ 10, -22 ≤ k ≤ 22, -23 ≤ l ≤ 23

Table S2. contd.

	**1b**^{NH3}	**2a**^{Cl}·H_2O	**3b**·toluene	**3b**^{Cl}·H_2O·toluene	**4a**·$CHCl_3$	**4b**^{Cl}·H_2O
**Reflections collected**	44752	36743	50594	95171	71288	79200
**Independent reflections**	5785 [R_{int} = 0.0399, R_{sigma} = 0.0213]	5300 [R_{int} = 0.0379, R_{sigma} = 0.0216]	11382 [R_{int} = 0.0354, R_{sigma} = 0.0317]	10870 [R_{int} = 0.0725, R_{sigma} = 0.0357]	8213 [R_{int} = 0.0506, R_{sigma} = 0.0242]	9686 [R_{int} = 0.0593, R_{sigma} = 0.0278]
**Data / restraints / parameters**	5785 / 42 / 486	5300 / 0 / 396	11382 / 53 / 779	10870 / 0 / 671	8213 / 4 / 530	9686 / 0 / 658
**Goodness-of-fit on F^2**	1.032	1.056	1.032	1.054	1.060	1.105
**Final R indexes [I>=2σ (I)]**	R_1 = 0.0210, wR_2 = 0.0502	R_1 = 0.0186, wR_2 = 0.0450	R_1 = 0.0327, wR_2 = 0.0838	R_1 = 0.0372, wR_2 = 0.0991	R_1 = 0.0300, wR_2 = 0.0718	R_1 = 0.0372, wR_2 = 0.0923
**Final R indexes [all data]**	R_1 = 0.0220, wR_2 = 0.0507	R_1 = 0.0205, wR_2 = 0.0458	R_1 = 0.0349, wR_2 = 0.0852	R_1 = 0.0420, wR_2 = 0.1026	R_1 = 0.0308, wR_2 = 0.0725	R_1 = 0.0414, wR_2 = 0.0947
**Largest diff. peak / hole [e $Å^{-3}$]**	0.34 / -0.39	0.80 / -0.66	0.78 / -0.53	0.86 / -1.18	1.67 / -1.11	2.31 / -2.27
**Flack parameter**	-	-	-0.011(4)	-	-	-

Highly disordered solvent; excluded with solvent mask from final refinements

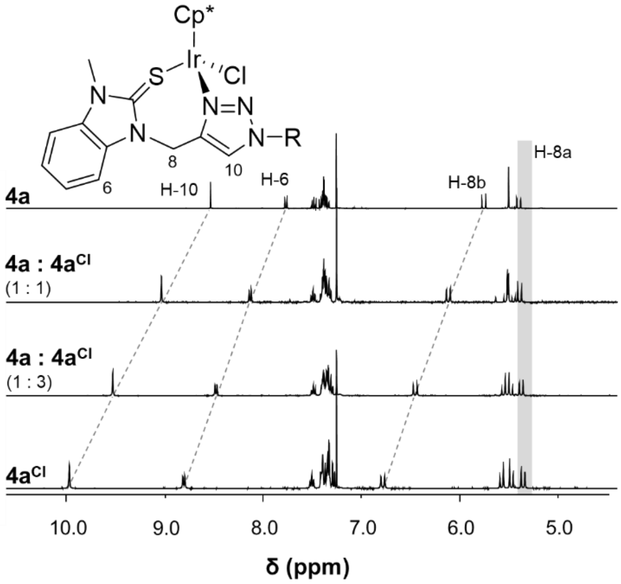

Figure S3. The aromatic regions of the ^{1}H NMR spectra of **4a** with its PF_6^- counterion and the chloride derivative **4a**^{Cl} and both compounds mixed at 1 : 1 and 1 : 3 ratios, recorded in $CDCl_3$.

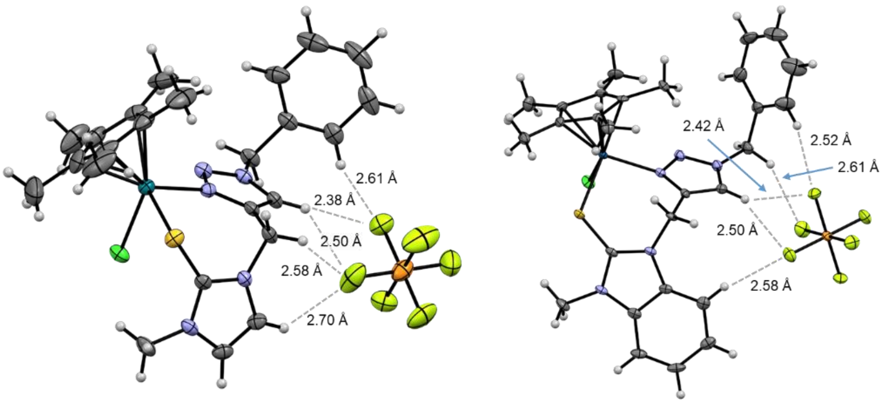

Figure S4. Interactions between the PF_6^- counterions and the complex cations in **3b** (left) and **4a** (right) with the C–H···F distances shown. Co-crystallized solvent molecules have been omitted for clarity and the structures are drawn at 50% probability level.

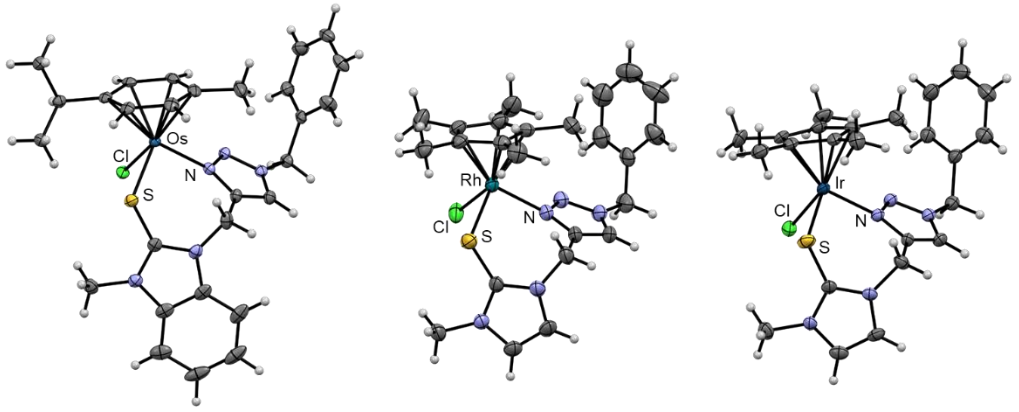

Figure S5. Molecular structures of one of the enantiomers found for **2a**^{Cl}, **3b**^{Cl}, and **4b**^{Cl}. Counterions and co-crystallized solvent molecules have been omitted for clarity and the structures are drawn at 50% probability level.

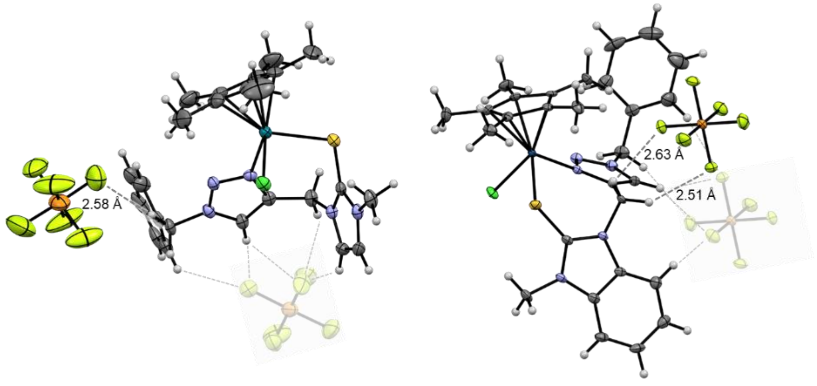

Figure S6. Secondary interactions between the PF_6^- counterions and the complex cations in **3b** (left) and **4a** (right). Co-crystallized solvent molecules have been omitted for clarity and the structures are drawn at 50% probability level.

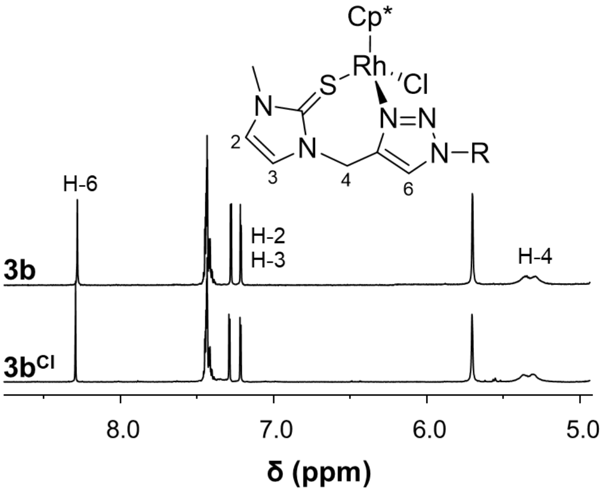

Figure S7. The aromatic regions of the ^{1}H NMR spectra of **3b** and **3b^{Cl}** recorded in MeOD.

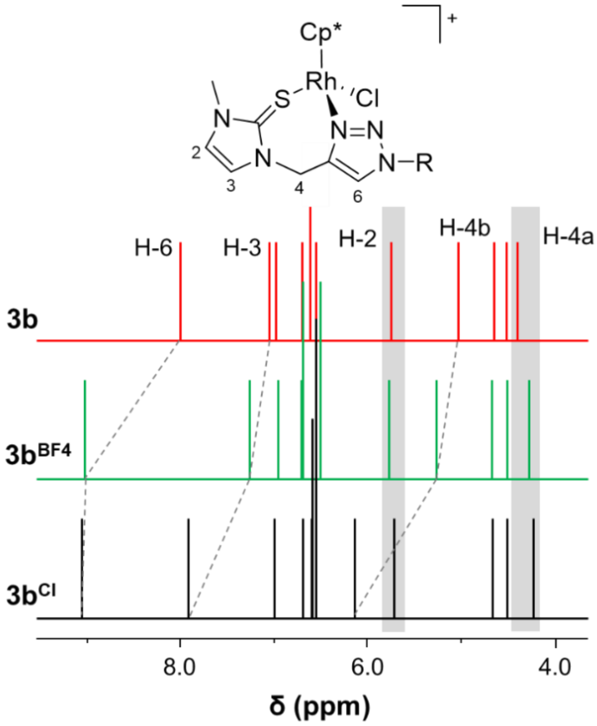

Figure S8. DFT calculations of ^{1}H NMR spectra of **3b**, **3b^{BF4}**, and **3b^{Cl}**.

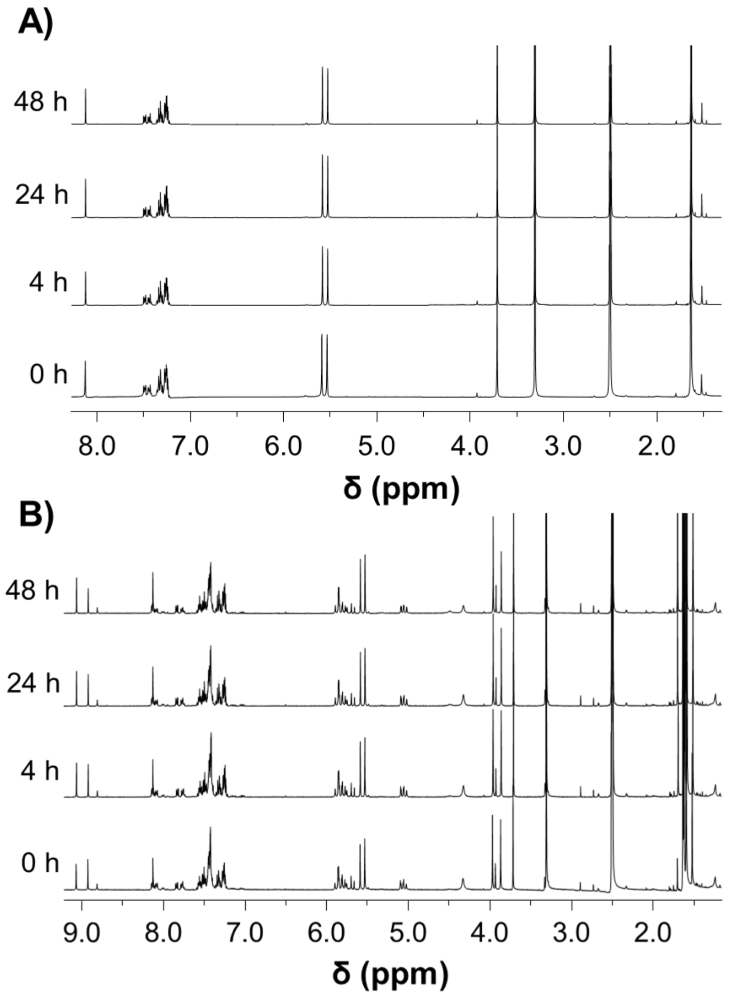

Figure S9. Stability of **4a** (A) and **4a**^{Cl} (B) in DMSO-d_6 investigated by ^{1}H NMR spectroscopy.

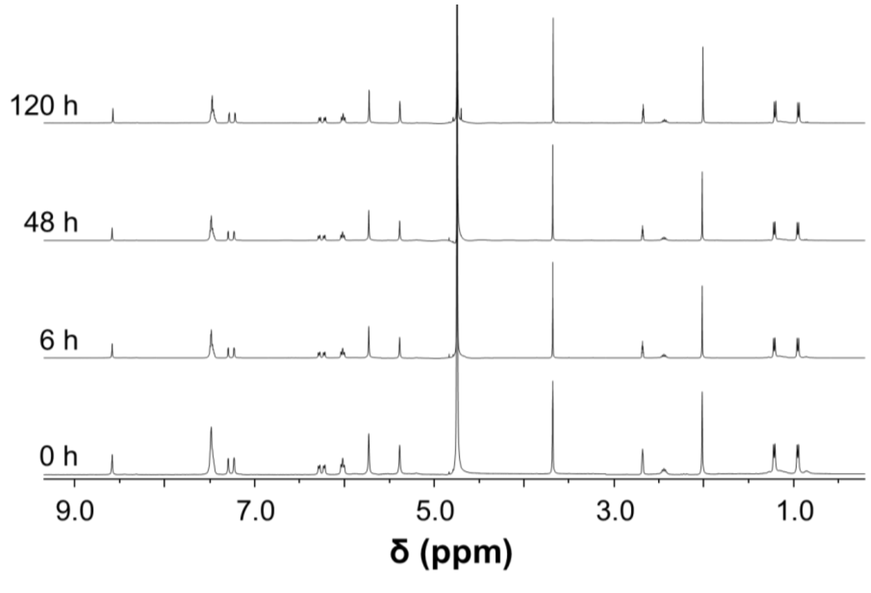

Figure S10. Stability of **1b** in 15% DMSO-d_6/D_2O investigated by ^{1}H NMR spectroscopy.

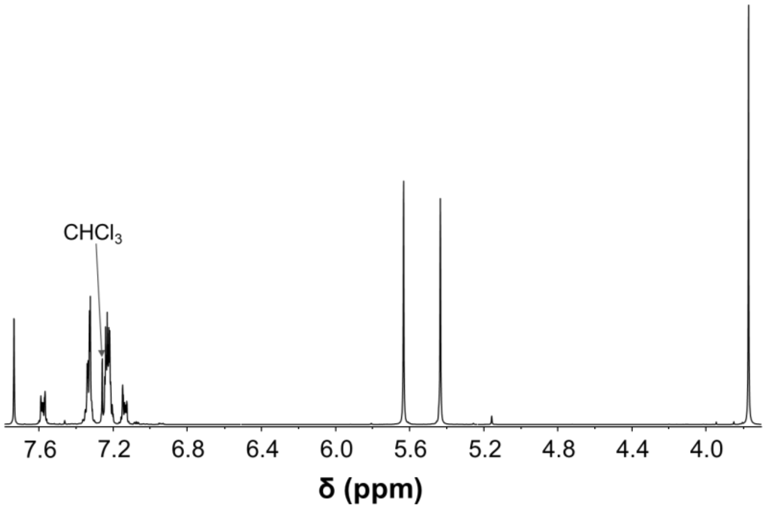

Figure S11. ^{1}H NMR spectrum of 1-[(1-benzyl-1,2,3-triazol-4-yl)methyl]-3-methylbenzimidazole-2-thione **a** recorded in $CDCl_3$.

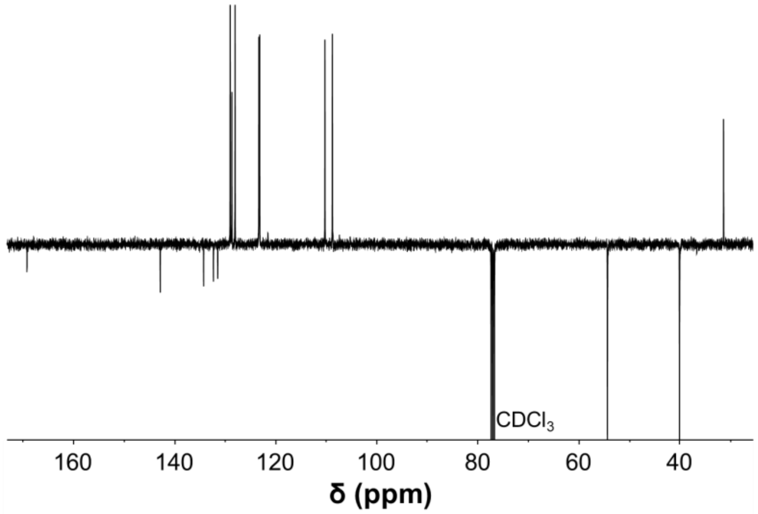

Figure S12. $^{13}C\{^{1}H\}$ DEPT-Q NMR spectrum of 1-[(1-benzyl-1,2,3-triazol-4-yl)methyl]-3-methylbenzimidazole-2-thione **a** recorded in $CDCl_3$.

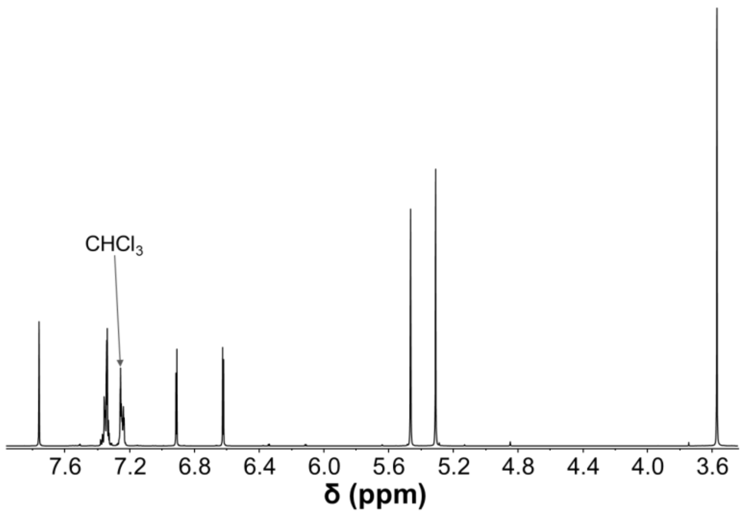

Figure S13. ^{1}H NMR spectrum of 1-[(1-benzyl-1,2,3-triazol-4-yl)methyl]-3-methylimidazole-2-thione **b** recorded in $CDCl_3$.

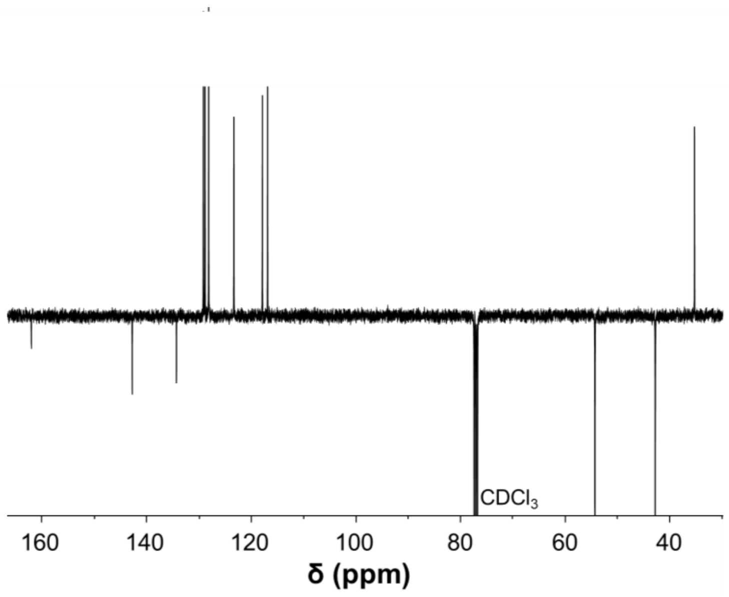

Figure S14. $^{13}C\{^{1}H\}$ DEPT-Q NMR spectrum of 1-[(1-benzyl-1,2,3-triazol-4-yl)methyl]-3-methylimidazole-2-thione **b** recorded in $CDCl_3$.

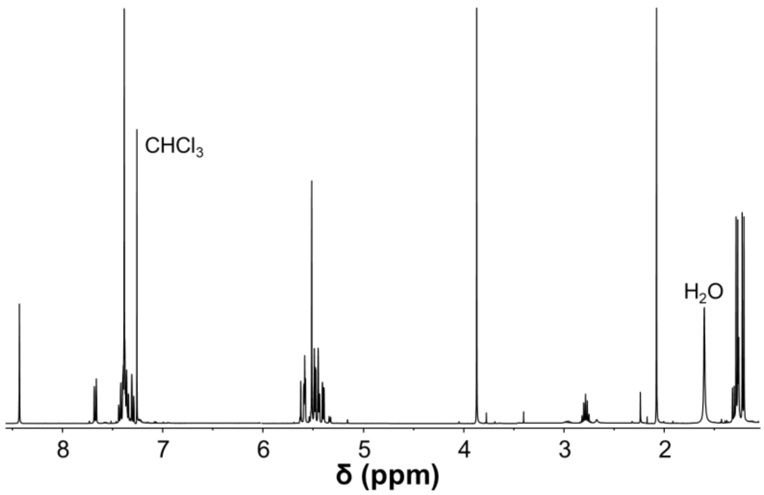

Figure S15. ^{1}H NMR spectrum of [chlorido{1-[(1-benzyl-1,2,3-triazol-4-yl-κ*N*)methyl]-3-methylbenzimidazole-2-thione-κ*S*}(η^{6}-*p*-cymene)ruthenium(II)] hexafluorophosphate **1a** recorded in $CDCl_3$.

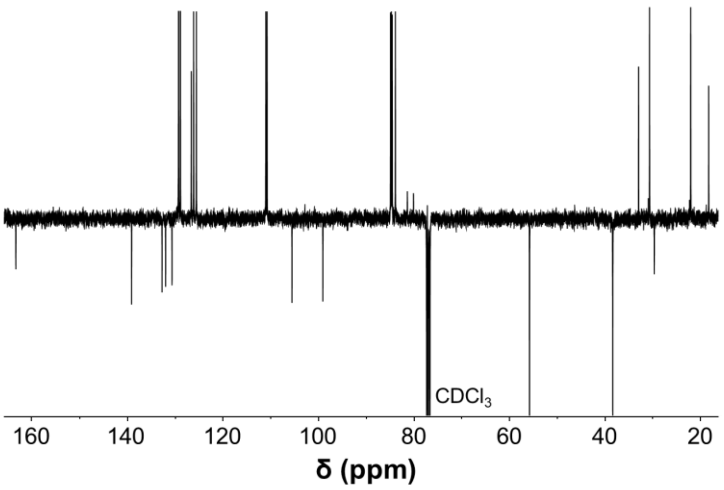

Figure S16. $^{13}C\{^{1}H\}$ DEPT-Q NMR spectrum of [chlorido{1-[(1-benzyl-1,2,3-triazol-4-yl-κ*N*)methyl]-3-methylbenzimidazole-2-thione-κ*S*}(η^{6}-*p*-cymene)ruthenium(II)] hexafluorophosphate **1a** recorded in $CDCl_3$.

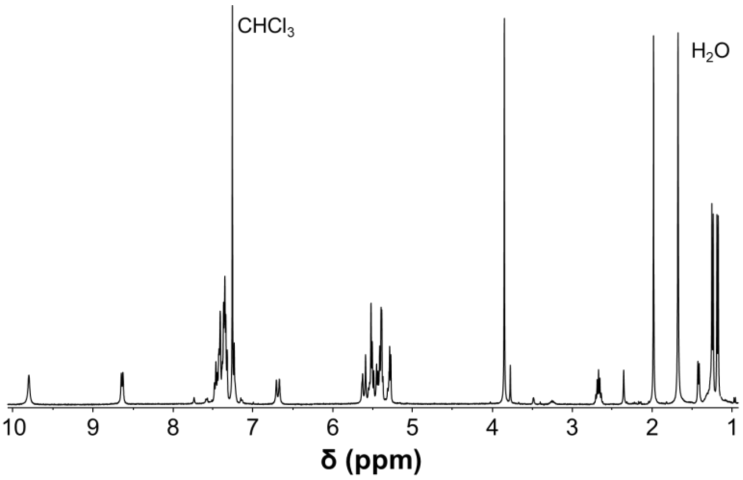

Figure S17. ^{1}H NMR spectrum of [chlorido{1-[(1-benzyl-1,2,3-triazol-4-yl-κ*N*)methyl]-3-methylbenzimidazole-2-thione-κ*S*}(η^{6}-*p*-cymene)ruthenium(II)] chloride **1a**^{Cl} recorded in $CDCl_3$.

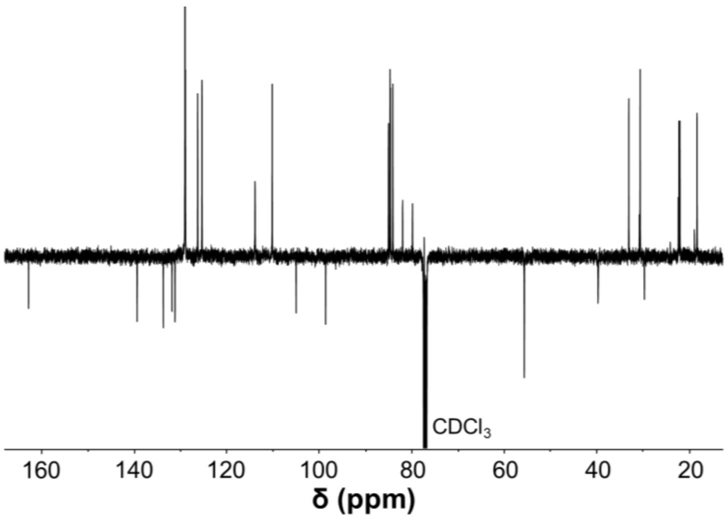

Figure S18. $^{13}C\{^{1}H\}$ DEPT-Q NMR spectrum of [chlorido{1-[(1-benzyl-1,2,3-triazol-4-yl-κ*N*)methyl]-3-methylbenzimidazole-2-thione-κ*S*}(η^{6}-*p*-cymene)ruthenium(II)] chloride **1a**^{Cl} recorded in $CDCl_3$.

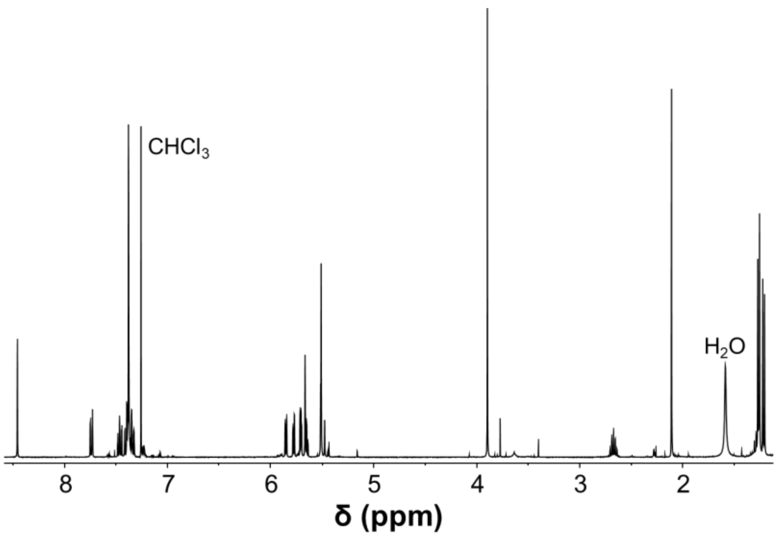

Figure S19. ^{1}H NMR spectrum of [chlorido{1-[(1-benzyl-1,2,3-triazol-4-yl-κ*N*)methyl]-3-methylbenzimidazole-2-thione-κ*S*}(η[6]-*p*-cymene)osmium(II)] hexafluorophosphate **2a** recorded in $CDCl_3$.

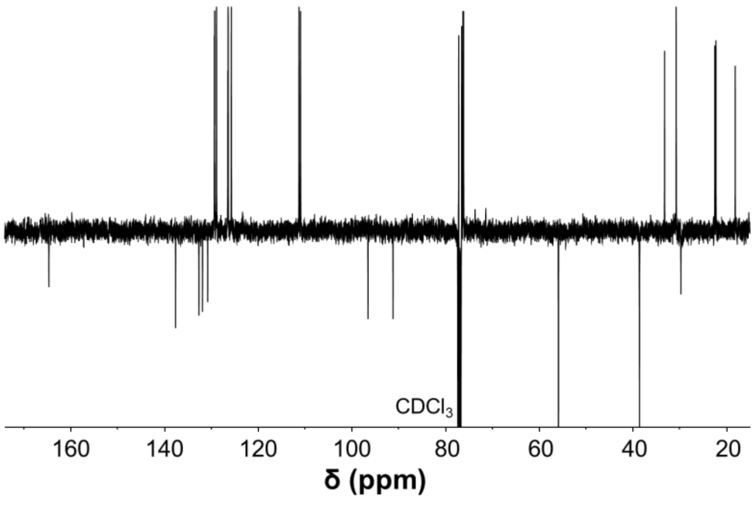

Figure S20. $^{13}C\{^{1}H\}$ DEPT-Q NMR spectrum of [chlorido{1-[(1-benzyl-1,2,3-triazol-4-yl-κ*N*)methyl]-3-methylbenzimidazole-2-thione-κ*S*}(η[6]-*p*-cymene)osmium(II)] hexafluorophosphate **2a** recorded in $CDCl_3$.

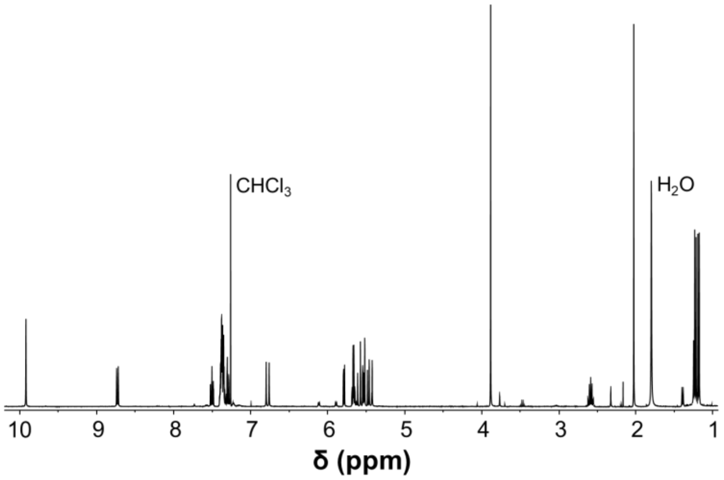

Figure S21. ^{1}H NMR spectrum of [chlorido{1-[(1-benzyl-1,2,3-triazol-4-yl-κN)methyl]-3-methylbenzimidazole-2-thione-κS}(η^{6}-*p*-cymene)osmium(II)] chloride **2a**^{Cl} recorded in $CDCl_3$.

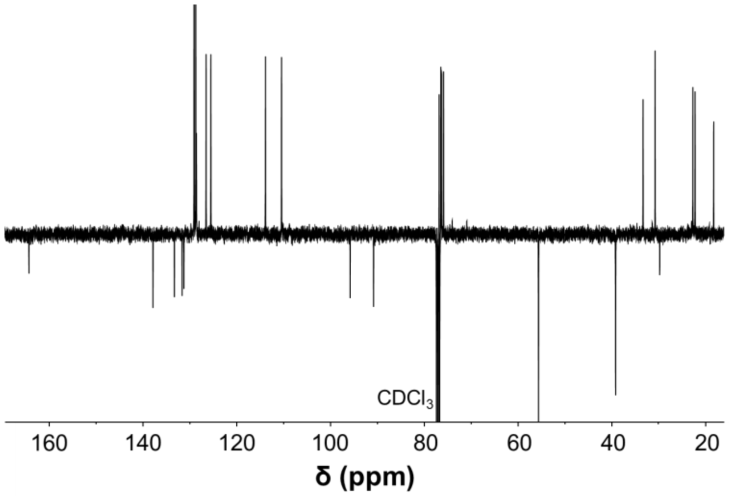

Figure S22. $^{13}C\{^{1}H\}$ DEPT-Q NMR spectrum of [chlorido{1-[(1-benzyl-1,2,3-triazol-4-yl-κN)methyl]-3-methylbenzimidazole-2-thione-κS}(η^{6}-*p*-cymene)osmium(II)] chloride **2a**^{Cl} recorded in $CDCl_3$.

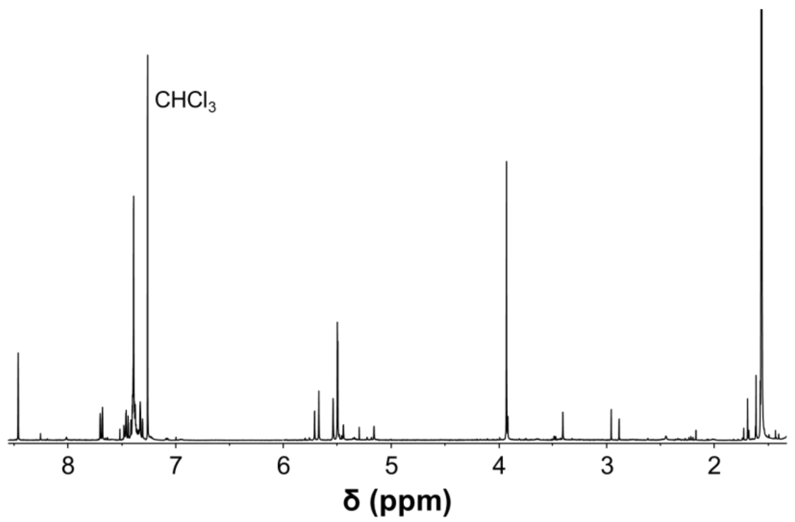

Figure S23. ^{1}H NMR spectrum of [chlorido{1-[(1-benzyl-1,2,3-triazol-4-yl-κN)methyl]-3-methylbenzimidazole-2-thione-κS}(η^5-pentamethylcyclopentadiene)rhodium(III)] hexafluorophosphate **3a** recorded in $CDCl_3$.

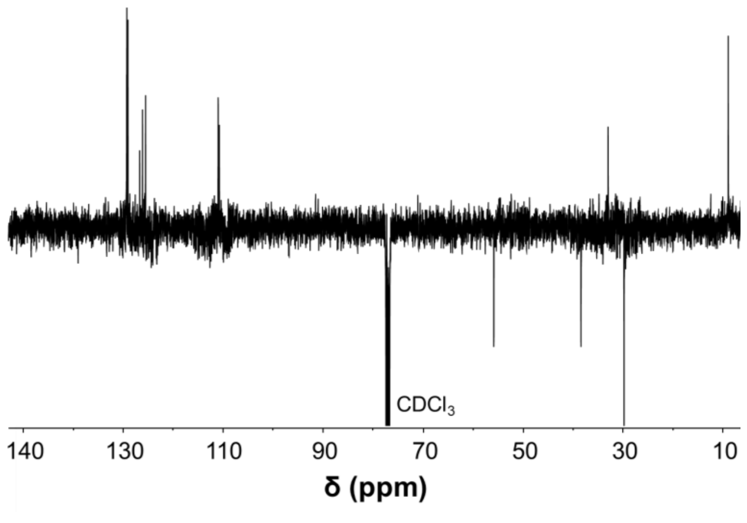

Figure S24. ^{13}C{^{1}H} DEPT-Q NMR spectrum of [chlorido{1-[(1-benzyl-1,2,3-triazol-4-yl-κN)methyl]-3-methylbenzimidazole-2-thione-κS}(η^5-pentamethylcyclopentadiene)rhodium(III)] hexafluorophosphate **3a** recorded in $CDCl_3$.

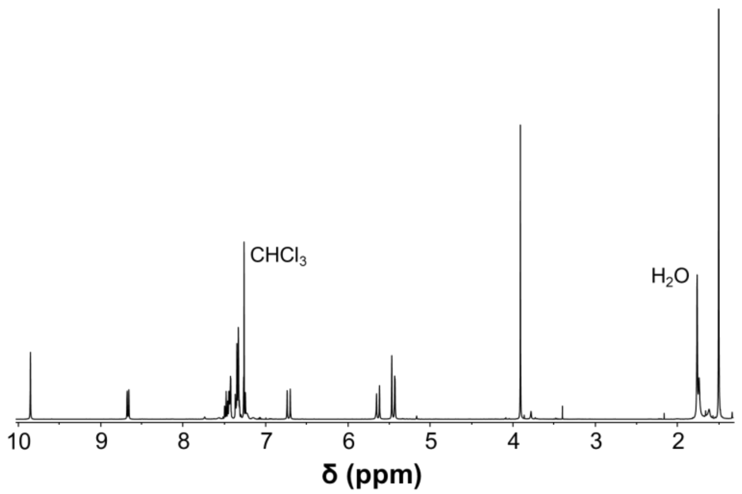

Figure S25. ^{1}H NMR spectrum of [chlorido{1-[(1-benzyl-1,2,3-triazol-4-yl-κN)methyl]-3-methylbenzimidazole-2-thione-κS}(η^{5}-pentamethylcyclopentadiene)rhodium(III)] chloride **3a**^{Cl} recorded in $CDCl_3$.

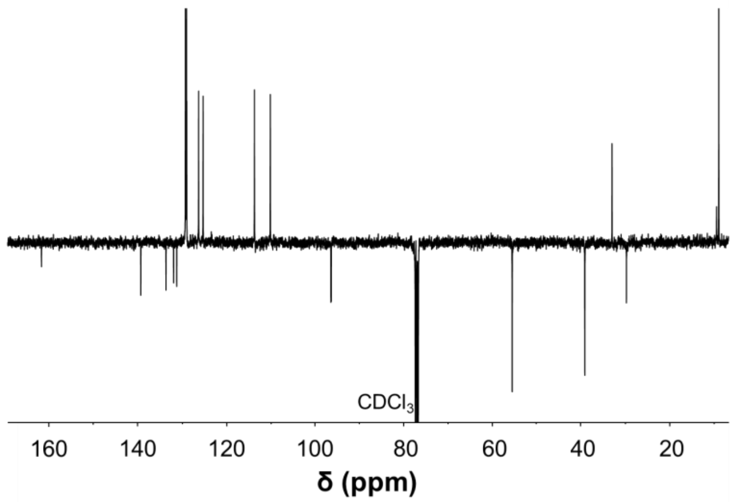

Figure S26. $^{13}C\{^{1}H\}$ DEPT-Q NMR spectrum of [chlorido{1-[(1-benzyl-1,2,3-triazol-4-yl-κN)methyl]-3-methylbenzimidazole-2-thione-κS}(η^{5}-pentamethylcyclopentadiene)rhodium(III)] chloride **3a**^{Cl} recorded in $CDCl_3$.

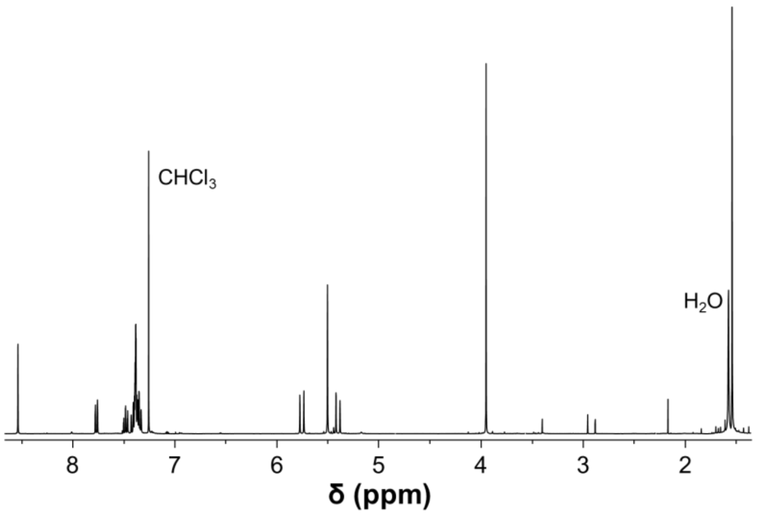

Figure S27. ^{1}H NMR spectrum of [chlorido{1-[(1-benzyl-1,2,3-triazol-4-yl-κN)methyl]-3-methylbenzimidazole-2-thione-κS}(η[5]-pentamethylcyclopentadiene)iridium(III)] hexafluorophosphate **4a** recorded in $CDCl_3$.

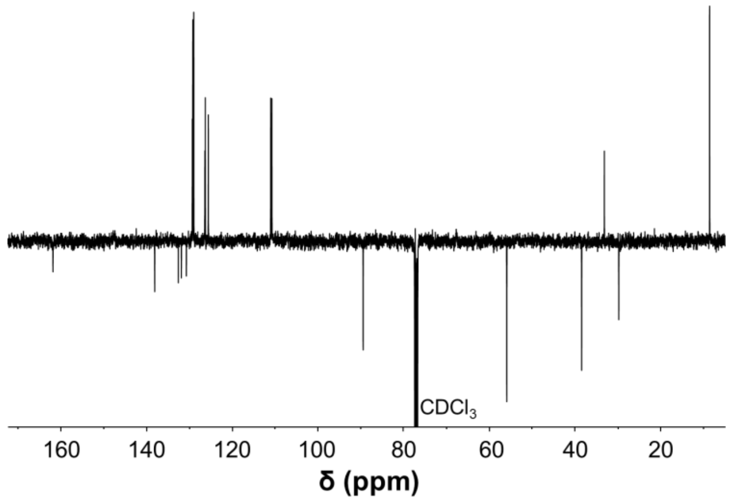

Figure S28. $^{13}C\{^1H\}$ DEPT-Q NMR spectrum of [chlorido{1-[(1-benzyl-1,2,3-triazol-4-yl-κN)methyl]-3-methylbenzimidazole-2-thione-κS}(η[5]-pentamethylcyclopentadiene)iridium(III)] hexafluorophosphate **4a** recorded in $CDCl_3$.

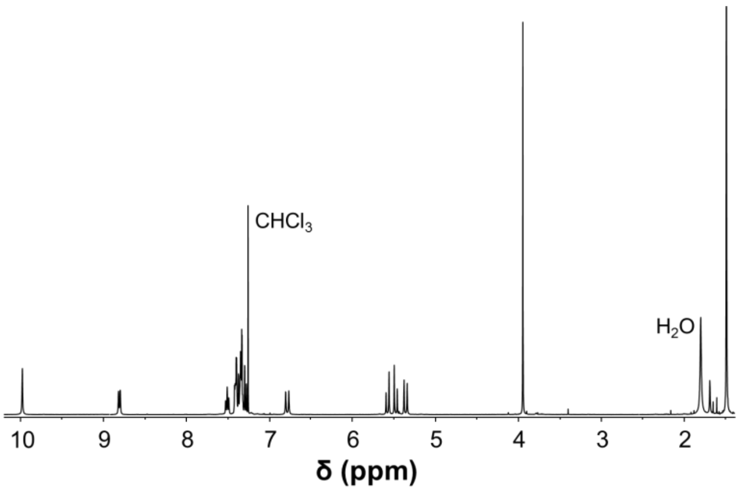

Figure S29. ^{1}H NMR spectrum of [chlorido{1-[(1-benzyl-1,2,3-triazol-4-yl-κN)methyl]-3-methylbenzimidazole-2-thione-κS}(η[5]-pentamethylcyclopentadiene)iridium(III)] chloride **4a**^{Cl} recorded in $CDCl_3$.

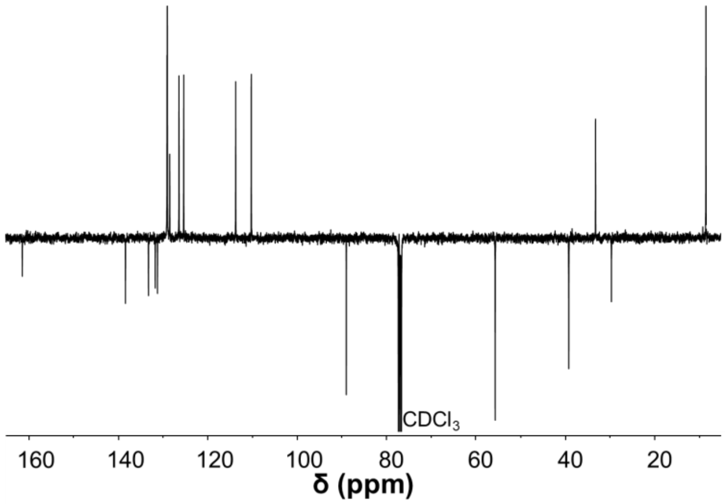

Figure S30. ^{13}C{^{1}H} DEPT-Q NMR spectrum of [chlorido{1-[(1-benzyl-1,2,3-triazol-4-yl-κN)methyl]-3-methylbenzimidazole-2-thione-κS}(η[5]-pentamethylcyclopentadiene)iridium(III)] chloride **4a**^{Cl} recorded in $CDCl_3$.

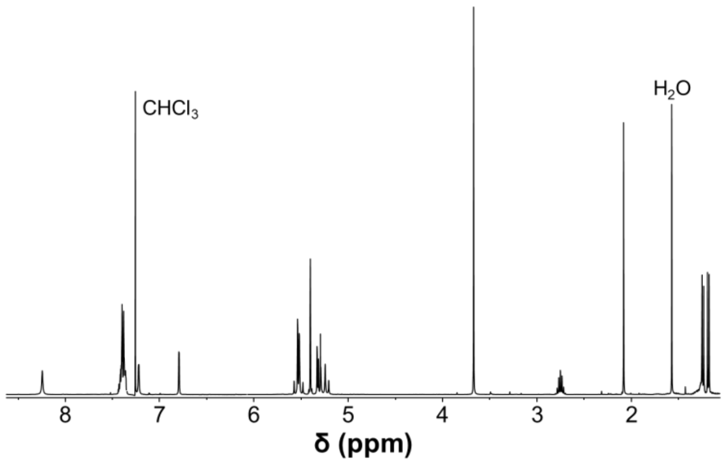

Figure S31. ^{1}H NMR spectrum of [chlorido{1-[(1-benzyl-1,2,3-triazol-4-yl-κN)methyl]-3-methylimidazole-2-thione-κS}(η^6-*p*-cymene)ruthenium(II)] hexafluorophosphate **1b** recorded in $CDCl_3$.

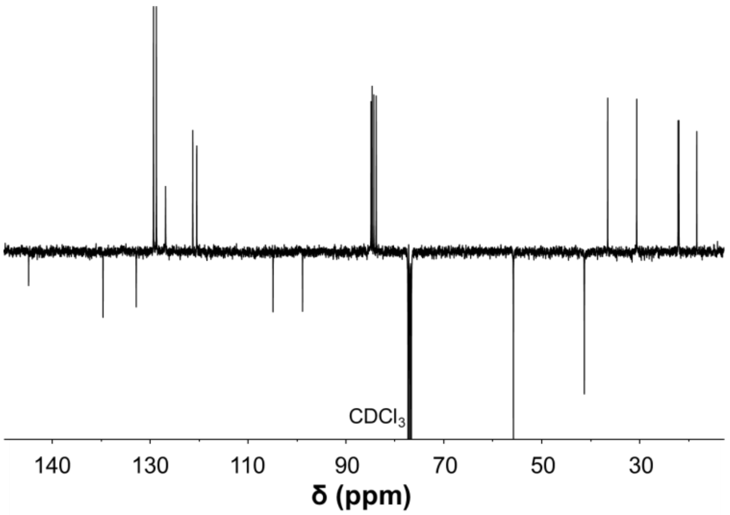

Figure S32. ^{13}C{^{1}H} DEPT-Q NMR spectrum of [chlorido{1-[(1-benzyl-1,2,3-triazol-4-yl-κN)methyl]-3-methylimidazole-2-thione-κS}(η^6-*p*-cymene)ruthenium(II)] hexafluorophosphate **1b** recorded in $CDCl_3$.

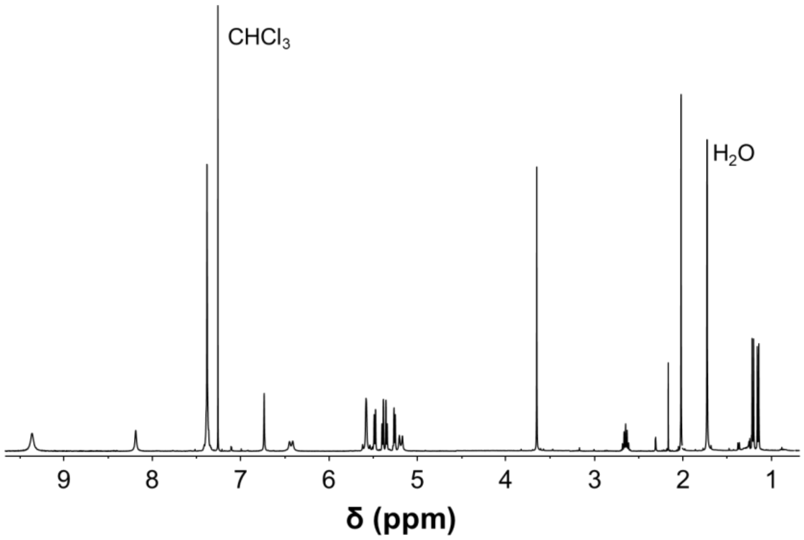

Figure S33. ^{1}H NMR spectrum of [chlorido{1-[(1-benzyl-1,2,3-triazol-4-yl-κN)methyl]-3-methylimidazole-2-thione-κS}(η^{6}-*p*-cymene)ruthenium(II)] chloride $\mathbf{1b}^{Cl}$ recorded in $CDCl_3$.

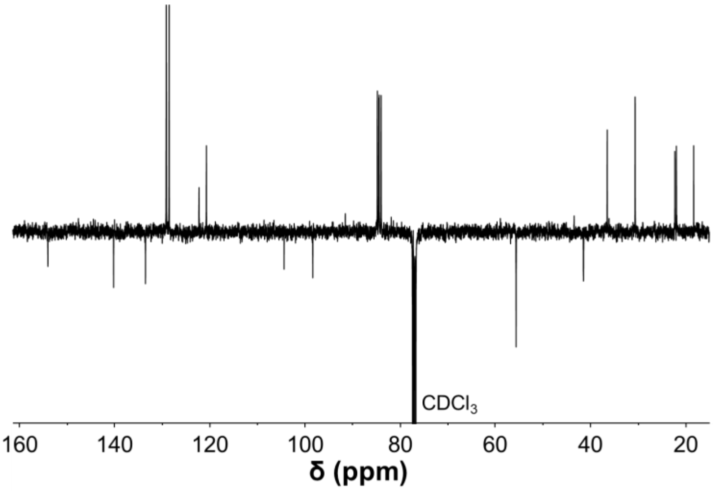

Figure S34. $^{13}C\{^{1}H\}$ DEPT-Q NMR spectrum of [chlorido{1-[(1-benzyl-1,2,3-triazol-4-yl-κN)methyl]-3-methylimidazole-2-thione-κS}(η^{6}-*p*-cymene)ruthenium(II)] chloride $\mathbf{1b}^{Cl}$ recorded in $CDCl_3$.

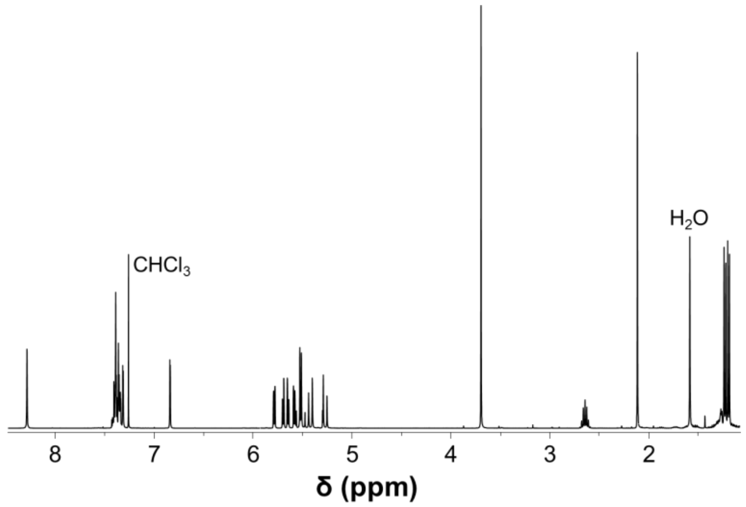

Figure S35. ^{1}H NMR spectrum of [chlorido{1-[(1-benzyl-1,2,3-triazol-4-yl-κN)methyl]-3-methylimidazole-2-thione-κS}(η[6]-*p*-cymene)osmium(II)] hexafluorophosphate **2b** recorded in $CDCl_3$.

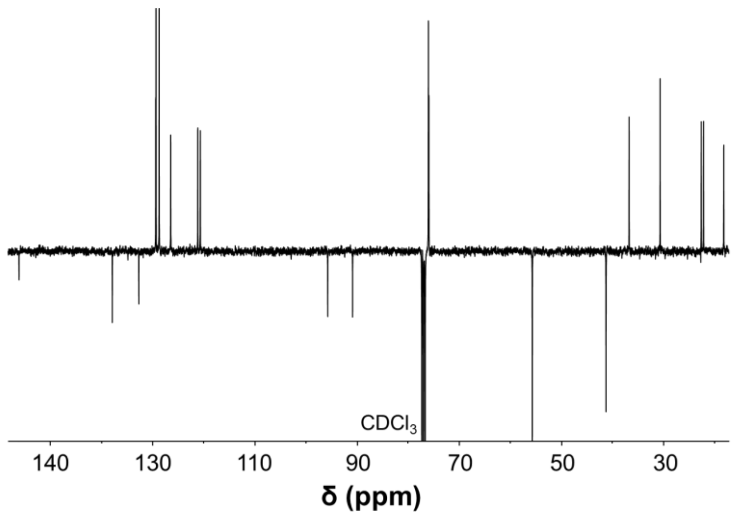

Figure S36. $^{13}C\{^{1}H\}$ DEPT-Q NMR spectrum of [chlorido{1-[(1-benzyl-1,2,3-triazol-4-yl-κN)methyl]-3-methylimidazole-2-thione-κS}(η[6]-*p*-cymene)osmium(II)] hexafluorophosphate **2b** recorded in $CDCl_3$.

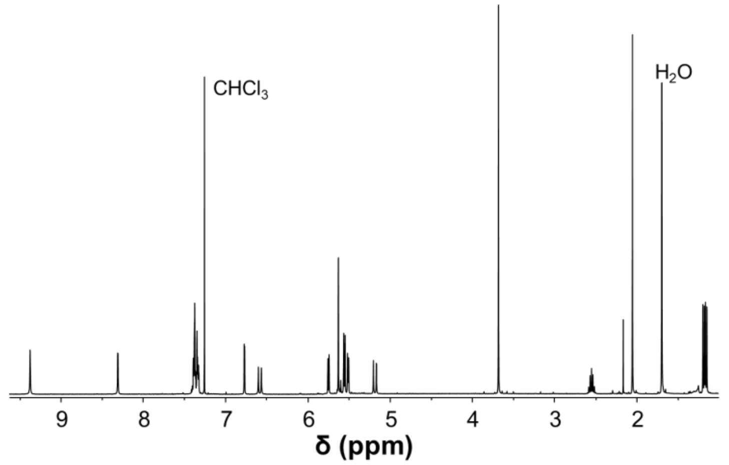

Figure S37. ^{1}H NMR spectrum of [chlorido{1-[(1-benzyl-1,2,3-triazol-4-yl-κN)methyl]-3-methylimidazole-2-thione-κS}(η[6]-*p*-cymene)osmium(II)] chloride **2b**[Cl] recorded in $CDCl_3$.

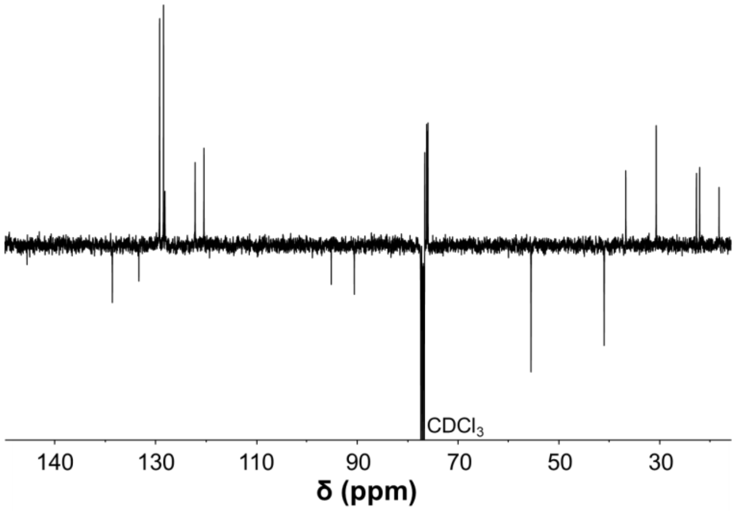

Figure S38. ^{13}C{^{1}H} DEPT-Q NMR spectrum of [chlorido{1-[(1-benzyl-1,2,3-triazol-4-yl-κN)methyl]-3-methylimidazole-2-thione-κS}(η[6]-*p*-cymene)osmium(II)] chloride **2b**[Cl] recorded in $CDCl_3$.

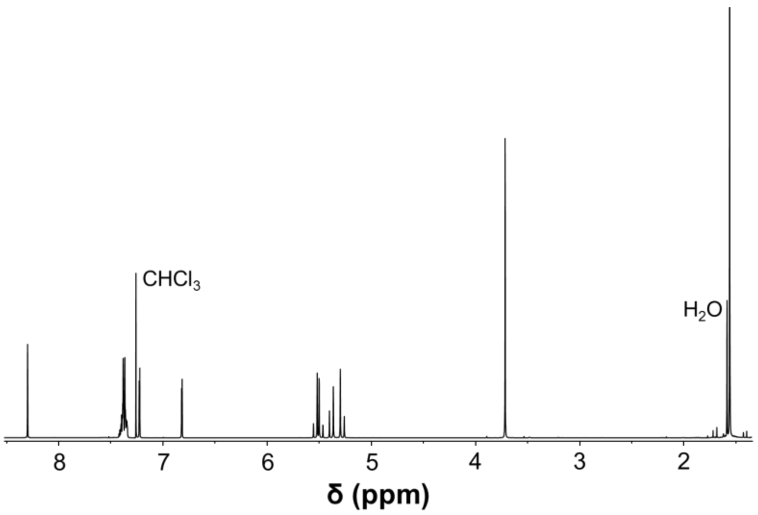

Figure S39. ^{1}H NMR spectrum of [chlorido{1-[(1-benzyl-1,2,3-triazol-4-yl-κN)methyl]-3-methylimidazole-2-thione-κS}(η^5-pentamethylcyclopentadiene)rhodium(III)] hexafluorophosphate **3b** recorded in CDCl$_3$.

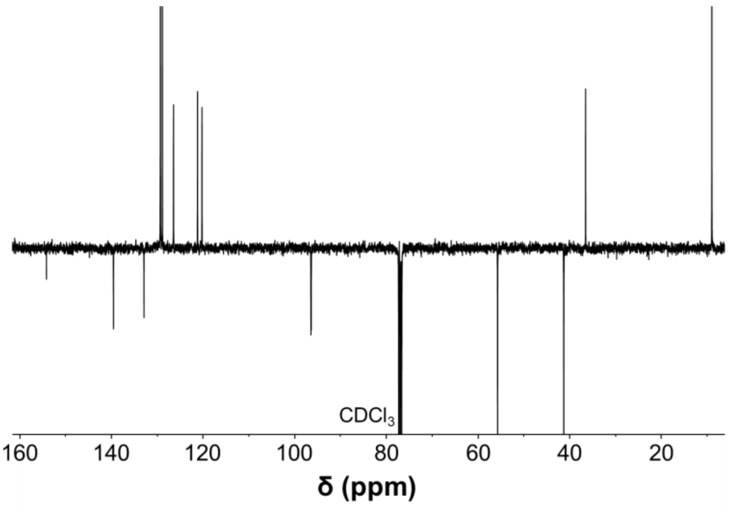

Figure S40. ^{13}C{^{1}H} DEPT-Q NMR spectrum of [chlorido{1-[(1-benzyl-1,2,3-triazol-4-yl-κN)methyl]-3-methylimidazole-2-thione-κS}(η^5-pentamethylcyclopentadiene)rhodium(III)] hexafluorophosphate **3b** recorded in CDCl$_3$.

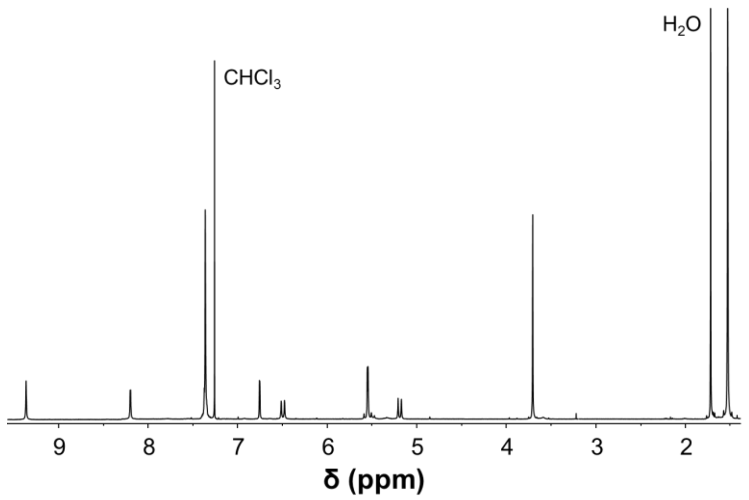

Figure S41. ^{1}H NMR spectrum of [chlorido{1-[(1-benzyl-1,2,3-triazol-4-yl-κN)methyl]-3-methylimidazole-2-thione-κS}(η^5-pentamethylcyclopentadiene)rhodium(III)] chloride **3b**^{Cl} recorded in $CDCl_3$.

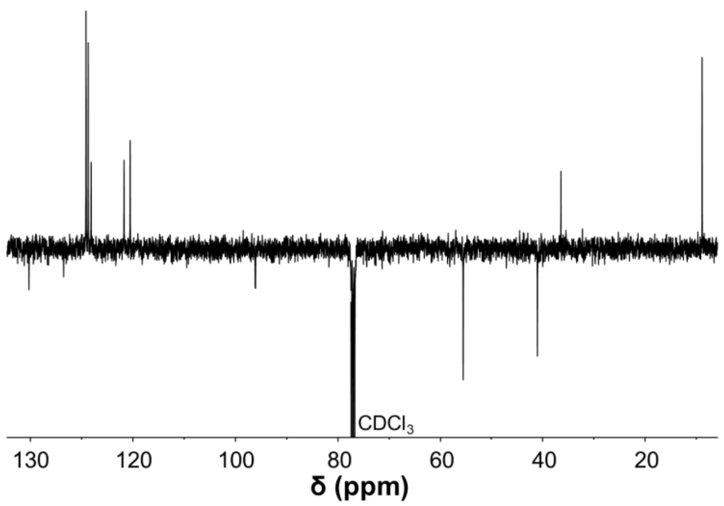

Figure S42. $^{13}C\{^{1}H\}$ DEPT-Q NMR spectrum of [chlorido{1-[(1-benzyl-1,2,3-triazol-4-yl-κN)methyl]-3-methylimidazole-2-thione-κS}(η^5-pentamethylcyclopentadiene)rhodium(III)] chloride **3b**^{Cl} recorded in $CDCl_3$.

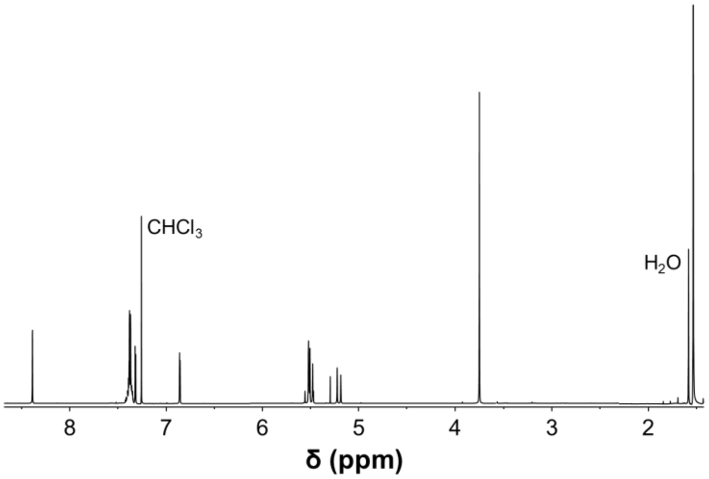

Figure S43. ^{1}H NMR spectrum of [chlorido{1-[(1-benzyl-1,2,3-triazol-4-yl-κN)methyl]-3-methylimidazole-2-thione-κS}(η^5-pentamethylcyclopentadiene)iridium(III)] hexafluorophosphate **4b** recorded in $CDCl_3$.

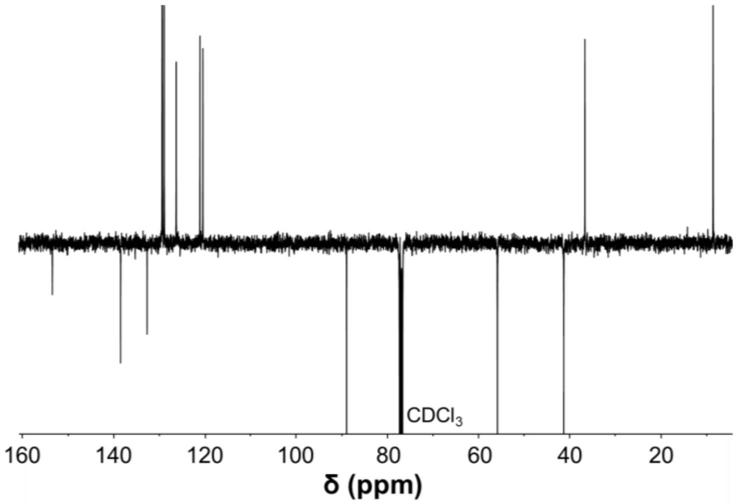

Figure S44. $^{13}C\{^{1}H\}$ DEPT-Q NMR spectrum of [chlorido{1-[(1-benzyl-1,2,3-triazol-4-yl-κN)methyl]-3-methylimidazole-2-thione-κS}(η^5-pentamethylcyclopentadiene)iridium(III)] hexafluorophosphate **4b** recorded in $CDCl_3$.

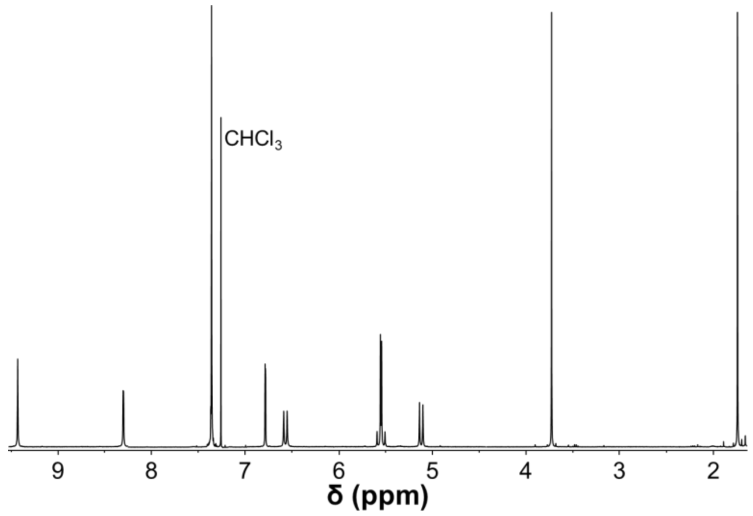

Figure S45. ^{1}H NMR spectrum of [chlorido{1-[(1-benzyl-1,2,3-triazol-4-yl-κN)methyl]-3-methylimidazole-2-thione-κS}(η[5]-pentamethylcyclopentadiene)iridium(III)] chloride **4b**^{Cl} recorded in $CDCl_3$.

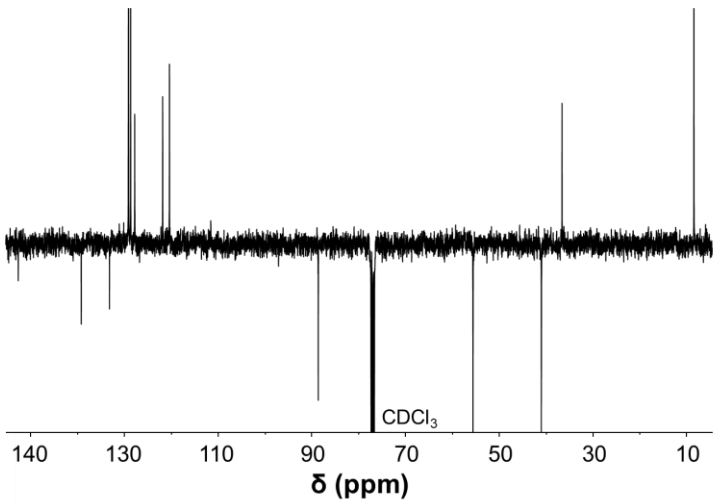

Figure S46. ^{13}C{^{1}H} DEPT-Q NMR spectrum of [chlorido{1-[(1-benzyl-1,2,3-triazol-4-yl-κN)methyl]-3-methylimidazole-2-thione-κS}(η[5]-pentamethylcyclopentadiene)iridium(III)] chloride **4b**^{Cl} recorded in $CDCl_3$.

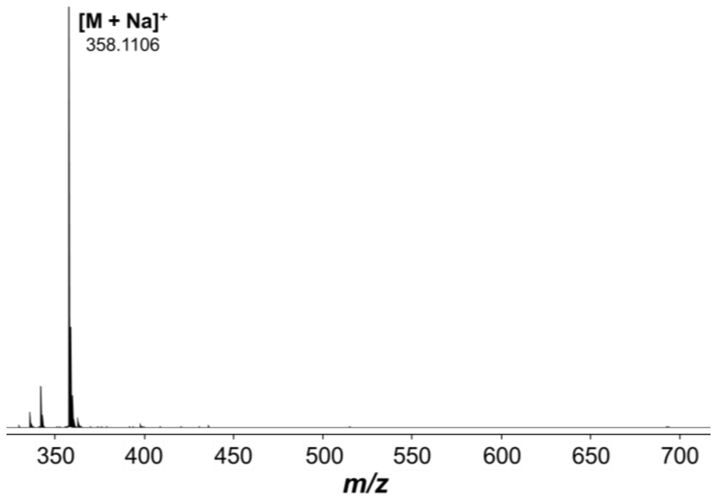

Figure S47. Electrospray ionization mass spectrum of 1-[(1-benzyl-1,2,3-triazol-4-yl)methyl]-3-methylbenzimidazole-2-thione **a**.

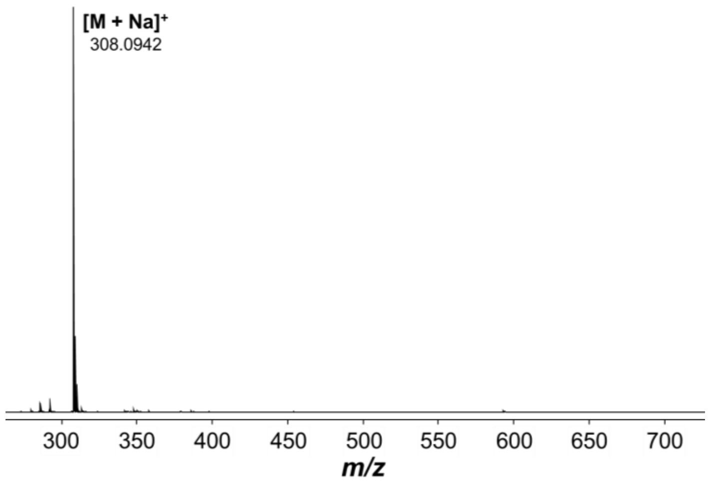

Figure S48. Electrospray ionization mass spectrum of 1-[(1-benzyl-1,2,3-triazol-4-yl)methyl]-3-methylimidazole-2-thione **b**.

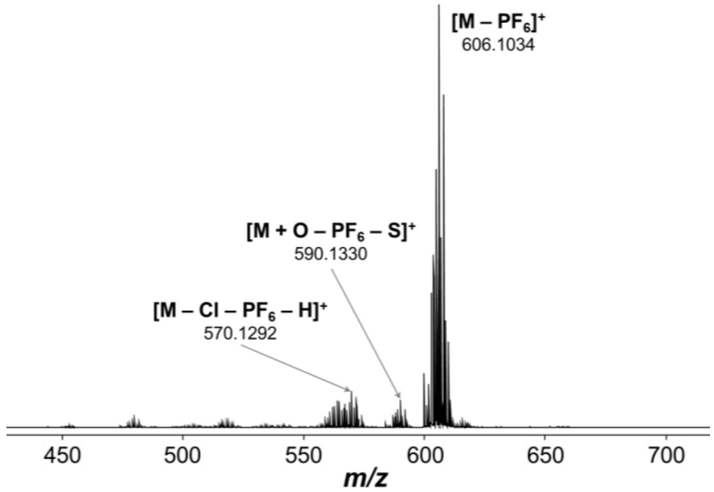

Figure S49. Electrospray ionization mass spectrum of [chlorido{1-[(1-benzyl-1,2,3-triazol-4-yl-κ*N*)methyl]-3-methylbenzimidazole-2-thione-κ*S*}(η^6-*p*-cymene)ruthenium(II)] hexafluorophosphate **1a**.

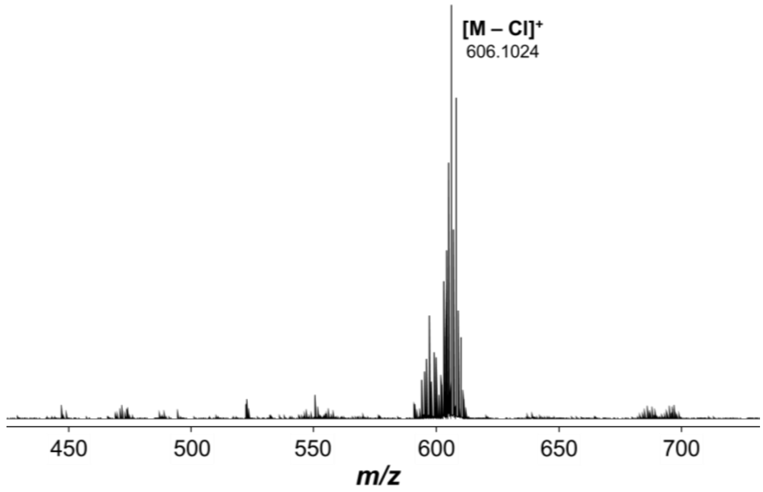

Figure S50. Electrospray ionization mass spectrum of [chlorido{1-[(1-benzyl-1,2,3-triazol-4-yl-κ*N*)methyl]-3-methylbenzimidazole-2-thione-κ*S*}(η^6-*p*-cymene)ruthenium(II)] chloride **1a**^{Cl}.

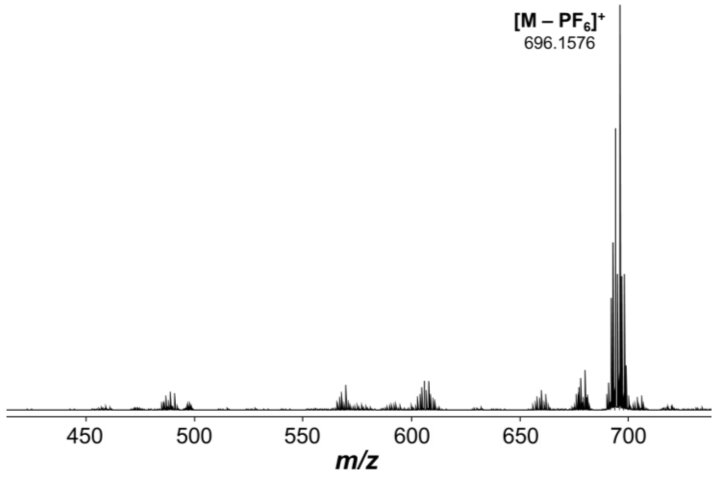

Figure S51. Electrospray ionization mass spectrum of [chlorido{1-[(1-benzyl-1,2,3-triazol-4-yl-κ*N*)methyl]-3-methylbenzimidazole-2-thione-κ*S*}(η^6-*p*-cymene)osmium(II)] hexafluorophosphate **2a**.

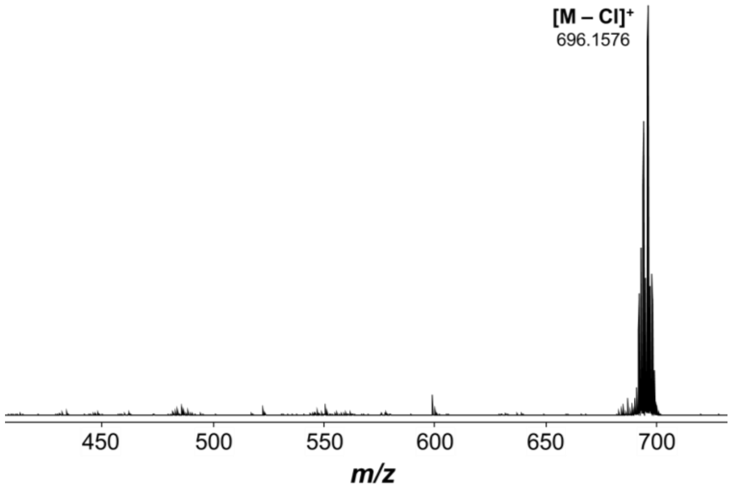

Figure S52. Electrospray ionization mass spectrum of [chlorido{1-[(1-benzyl-1,2,3-triazol-4-yl-κN)methyl]-3-methylbenzimidazole-2-thione-κS}(η^6-*p*-cymene)osmium(II)] chloride **2a**^{Cl}.

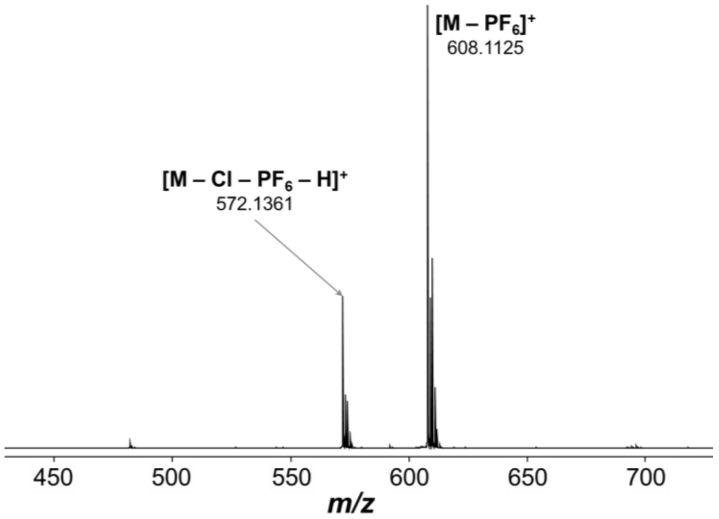

Figure S53. Electrospray ionization mass spectrum of [chlorido{1-[(1-benzyl-1,2,3-triazol-4-yl-κN)methyl]-3-methylbenzimidazole-2-thione-κS}(η[5]-pentamethylcyclopentadiene)rhodium(III)] hexafluorophosphate **3a**.

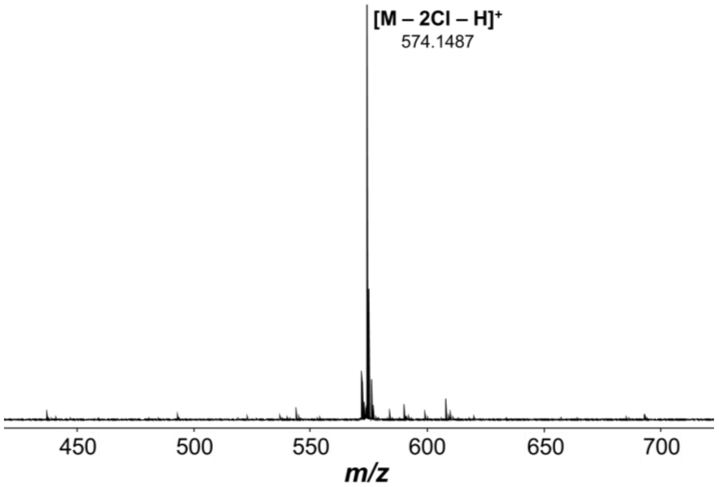

Figure S54. Electrospray ionization mass spectrum of [chlorido{1-[(1-benzyl-1,2,3-triazol-4-yl-κN)methyl]-3-methylbenzimidazole-2-thione-κS}(η[5]-pentamethylcyclopentadiene)rhodium(III)] chloride **3a**^{Cl}.

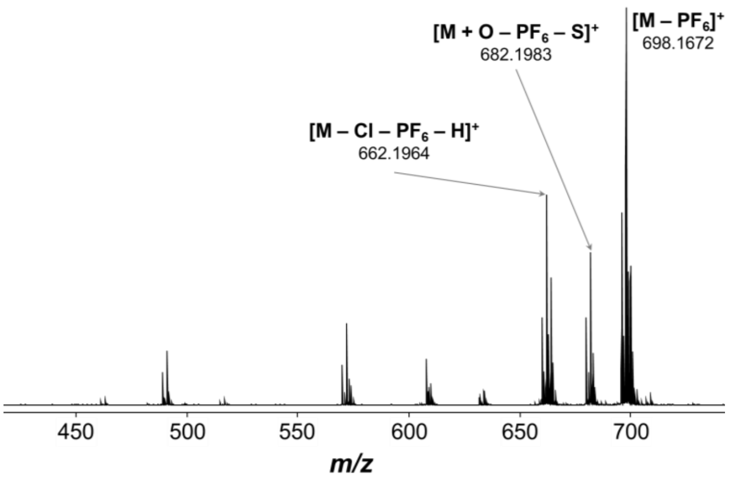

Figure S55. Electrospray ionization mass spectrum of [chlorido{1-[(1-benzyl-1,2,3-triazol-4-yl-κN)methyl]-3-methylbenzimidazole-2-thione-κS}(η^5-pentamethylcyclopentadiene)iridium(III)] hexafluorophosphate **4a**.

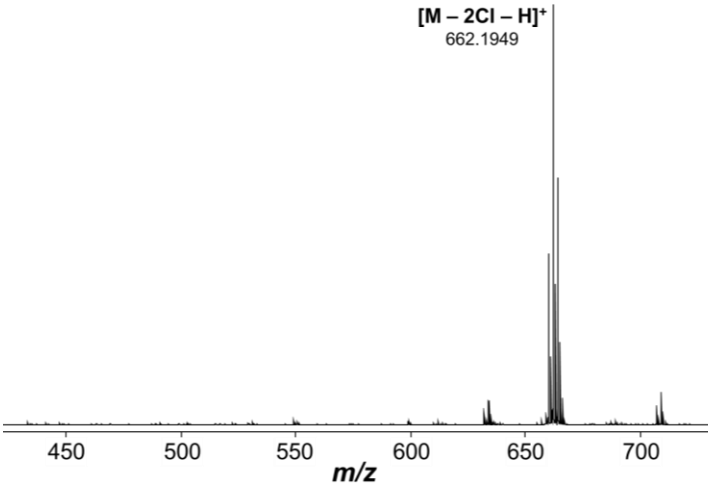

Figure S56. Electrospray ionization mass spectrum of [chlorido{1-[(1-benzyl-1,2,3-triazol-4-yl-κN)methyl]-3-methylbenzimidazole-2-thione-κS}(η^5-pentamethylcyclopentadiene)iridium(III)] chloride **4a**^{Cl}.

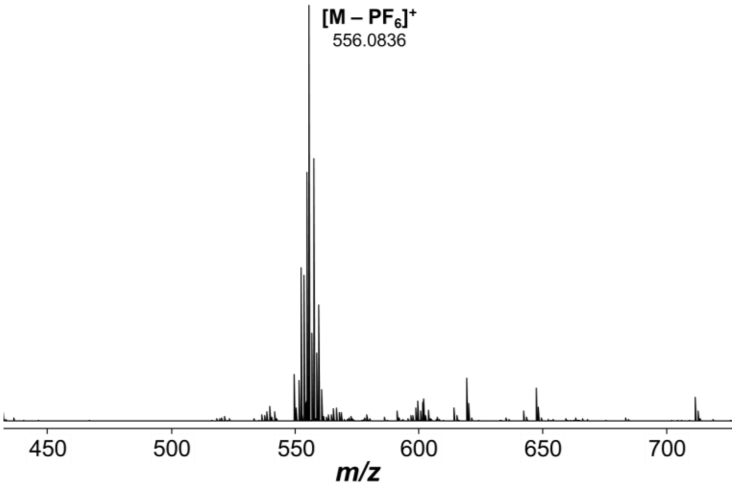

Figure S57. Electrospray ionization mass spectrum of [chlorido{1-[(1-benzyl-1,2,3-triazol-4-yl-κN)methyl]-3-methylimidazole-2-thione-κS}(η[6]-*p*-cymene)ruthenium(II)] hexafluorophosphate **1b**.

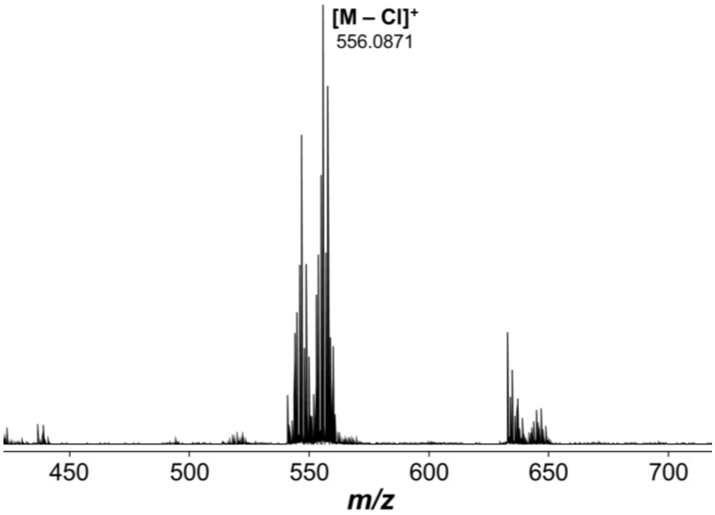

Figure S58. Electrospray ionization mass spectrum of [chlorido{1-[(1-benzyl-1,2,3-triazol-4-yl-κN)methyl]-3-methylimidazole-2-thione-κS}(η[6]-*p*-cymene)ruthenium(II)] chloride **1b^{Cl}**.

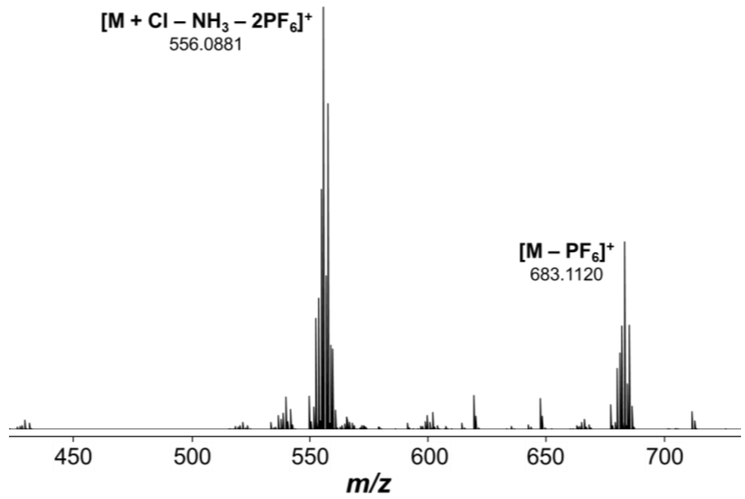

Figure S59. Electrospray ionization mass spectrum of [ammine{1-[(1-benzyl-1,2,3-triazol-4-yl-κN)methyl]-3-methylimidazole-2-thione-κS}(η[6]-*p*-cymene)ruthenium(II)] hexafluorophosphate **1b**^{NH3}.

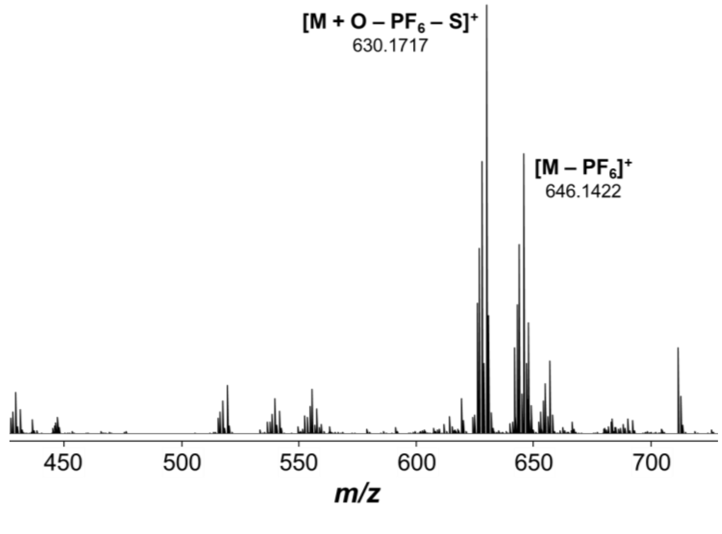

Figure S60. Electrospray ionization mass spectrum of [chlorido{1-[(1-benzyl-1,2,3-triazol-4-yl-κN)methyl]-3-methylimidazole-2-thione-κS}(η[6]-*p*-cymene)osmium(II)] hexafluorophosphate **2b**.

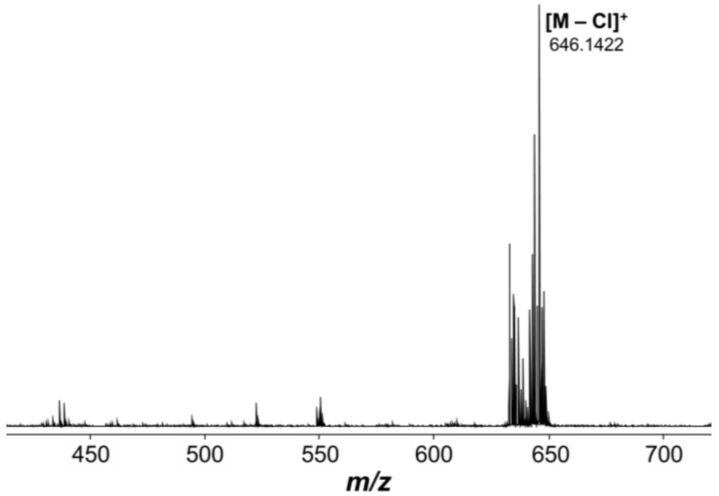

Figure S61. Electrospray ionization mass spectrum of [chlorido{1-[(1-benzyl-1,2,3-triazol-4-yl-κN)methyl]-3-methylimidazole-2-thione-κS}(η^6-*p*-cymene)osmium(II)] chloride **2b**$^{\mathbf{Cl}}$.

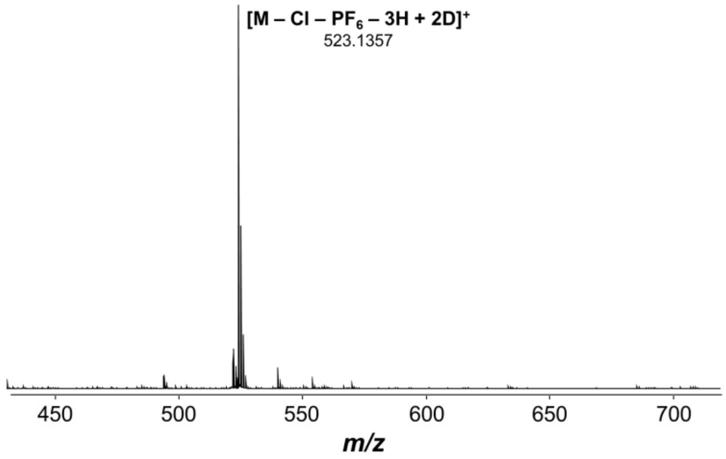

Figure S62. Electrospray ionization mass spectrum of [chlorido{1-[(1-benzyl-1,2,3-triazol-4-yl-κN)methyl]-3-methylimidazole-2-thione-κS}(η^5-pentamethylcyclopentadiene)rhodium(III)] hexafluorophosphate **3b**.

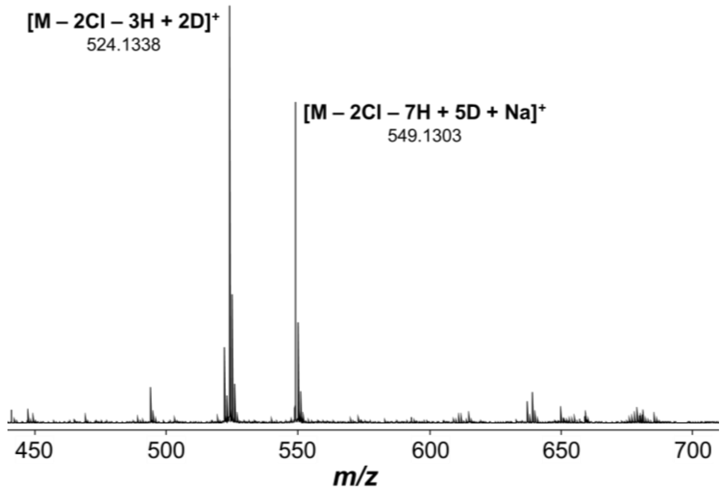

Figure S63. Electrospray ionization mass spectrum of [chlorido{1-[(1-benzyl-1,2,3-triazol-4-yl-κN)methyl]-3-methylimidazole-2-thione-κS}(η^5-pentamethylcyclopentadiene)rhodium(III)] chloride **3b**^{Cl}.

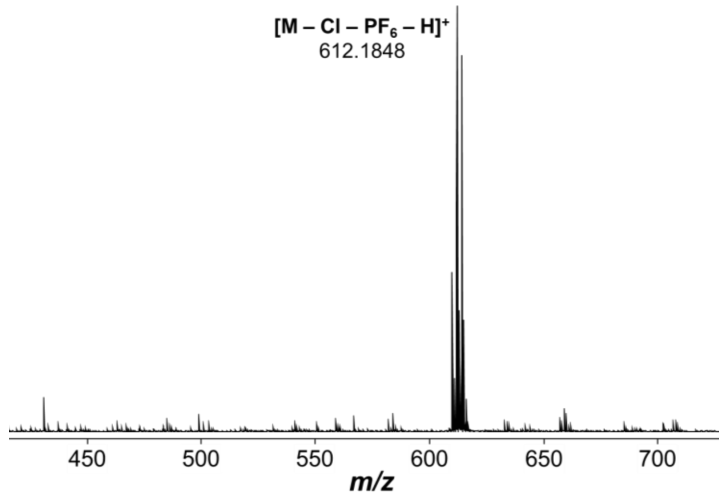

Figure S64. Electrospray ionization mass spectrum of [chlorido{1-[(1-benzyl-1,2,3-triazol-4-yl-κN)methyl]-3-methylimidazole-2-thione-κS}(η^5-pentamethylcyclopentadiene)iridium(III)] hexafluorophosphate **4b**.

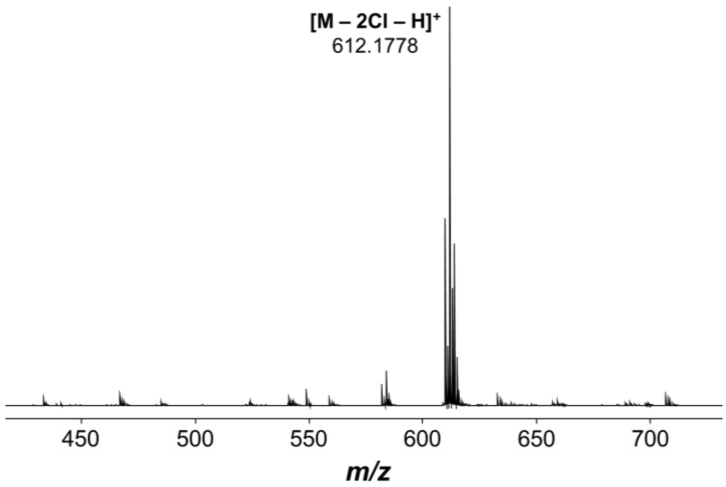

Figure S65. Electrospray ionization mass spectrum of [chlorido{1-[(1-benzyl-1,2,3-triazol-4-yl-κN)methyl]-3-methylimidazole-2-thione-κS}(η^5-pentamethylcyclopentadiene)iridium(III)] chloride **4b**^{Cl}.
